# Overexpression of an apple LysM-containing protein gene, *MdCERK1–2*, confers improved resistance to the pathogenic fungus, *Alternaria alternata*, in *Nicotiana benthamiana*

**DOI:** 10.1186/s12870-020-02361-z

**Published:** 2020-04-08

**Authors:** Qiming Chen, Chaohua Dong, Xiaohong Sun, Yugang Zhang, Hongyi Dai, Suhua Bai

**Affiliations:** 1 College of Life Sciences, Key Laboratory of Plant Biotechnology of Shandong Province, Qingdao Agricultural University, 700 Changcheng Road, Qingdao, 266109 China; 2 Qingdao Key Laboratory of Genetic Improvement and Breeding in Horticultural Plants, Qingdao Agricultural University, Qingdao, 266109 China; 3 Shandong Province Key Laboratory of Applied Mycology, Qingdao, 266109 China; 4 College of Horticulture, Qingdao Agricultural University, Qingdao, 266109 China

**Keywords:** Fungal pathogen, LysM-containing protein, *Malus × domestica*, *Nicotiana benthamiana*, Plant immunity

## Abstract

**Background:**

Lysin motif (LysM)-containing proteins are involved in the recognition of fungal and bacterial pathogens. However, few studies have reported on their roles in the defense responses of woody plants against pathogens. A previous study reported that the apple *MdCERK1* gene was induced by chitin and *Rhizoctonia solani*, and its protein can bind to chitin. However, its effect on defense responses has not been investigated.

**Results:**

In this study, a new apple *CERK* gene, designated as *MdCERK1–2*, was identified. It encodes a protein that shares high sequence identity with the previously reported MdCERK1 and AtCERK1. Its chitin binding ability and subcellular location are similar to MdCERK1 and AtCERK1, suggesting that MdCERK1–2 may play a role in apple immune defense responses as a pattern recognition receptor (PRR). *MdCERK1–2* expression in apple was induced by 2 fungal pathogens, *Botryosphaeria dothidea* and *Glomerella cingulate,* but not by the bacterial pathogen, *Erwinia amylovora*, indicating that *MdCERK1–2* is involved in apple anti-fungal defense responses. Further functional analysis by heterologous overexpression (OE) in *Nicotiana benthamiana* (*Nb*) demonstrated that *MdCERK1–2* OE improved *Nb* resistance to the pathogenic fungus, *Alternaria alternata*. H_2_O_2_ accumulation and callose deposition increased after *A. alternata* infection in *MdCERK1–2* OE plants compared to wild type (WT) and empty vector (EV)-transformed plants. The induced expression of *NbPAL4* by *A. alternata* significantly (*p* < 0.01, *n* = 4) increased in *MdCERK1–2* OE plants. Other tested genes, including *NbNPR1*, *NbPR1a*, *NbERF1*, and *NbLOX1*, did not exhibit significant changes after *A. alternata* infection in OE plants compared to EV or WT plants. OE plants also accumulated more polyphenols after *A. alternata* infection.

**Conclusions:**

Heterologous *MdCERK1–2* OE affects multiple defense responses in *Nb* plants and increased their resistance to fungal pathogens. This result also suggests that *MdCERK1–2* is involved in apple defense responses against pathogenic fungi.

## Background

Plants are constantly subjected to attack from various pathogenic microorganisms. To fight against pathogen infection, plants have developed sophisticated immune systems that ward off pathogens and protect the plant from infection. During pathogen infection, plants first detect pathogen-associated molecular patterns (PAMPs) via pattern recognition receptors (PRRs), and then initiate a series of rapid PAMP-triggered immunity (PTI) responses to limit the proliferation and spread of pathogens [1]. Thus, PRRs are a pivotal component of innate plant immune systems. To date, some plant PRRs have been identified [2], including FLAGELLIN SENSING 2 (FLS2) [3], LYM1/3 [4], ELONGATION FACTOR-TU RECEPTOR (EFR) [5], PEP1 RECEPTOR 1 (PEPR1), PEPR2 [6–8], CHITIN ELICITOR RECEPTOR KINASE1 (CERK1) [9–11], and CHITIN ELICITOR BINDING PROTEIN (CEBiP) [12], which perceive the flg22 bacterial flagellin epitope, bacterial peptidoglycan, EF-Tu epitope elf18, plant elicitor peptides (Peps) released during pathogen infection, and chitin, respectively. Upon binding to corresponding ligands, these PRRs initiate downstream defense responses, such as a transient influx of calcium ions, ROS bursts, MAPKs activation, and the increased expression of pathogenesis-related (PR) protein genes.

Lysin motif (LysM)-containing proteins are involved in the recognition of fungal and bacterial pathogens as PRRs. They were first identified from bacteria with the ability to bind to peptidoglycan (PGN) [13]. OsCEBiP and AtCERK1 are well-studied plant LysM-containing proteins that recognize chitin, a representative PAMP of pathogenic fungi that initiates downstream immune responses [14]. AtCERK1 contains a 3-LysM ectodomain and intracellular Ser/Thr kinase region, and is an essential receptor for chitin elicitor signaling in *Arabidopsis thaliana* [9]. Two AtCERK1s form a sandwich-type heterotetramer complex with a LysM-containing receptor-like kinase (LYK), LYK5, another LysM-containing protein with higher chitin binding affinity that is indispensable for chitin-induced AtCERK1 phosphorylation and immune responses in *Arabidopsis* [15]. Rice CERK1 (OsCERK1) contains 2 LysMs, a transmembrane region, and an intracellular Ser/Thr kinase region that is essential for the transduction of immune signals [16]. Unlike AtCERK1, OsCERK1 does not directly bind to chitin. Instead, it recognizes chitin by forming a sandwich-like heterotetramer complex receptor with OsCEBiP, another LysM-containing protein with the ability to bind to chitin that elicits downstream immune responses in rice [12, 17]. OsCEBiP lacks an intracellular kinase domain and depends on OsCERK1 to transmit signals to plant cells.

In addition to the aforementioned LysM proteins, many other members of this family are involved in pathogen recognition. The *Arabidopsis* genome encodes 5 LYKs: LYK1/CERK1 and LYK2 through 5 [11, 18]. LYK3 is involved in chitin signaling as a negative regulator in the regulation of Arabidopsis resistance to *Botrytis cinerea* and *Pectobacterium carotovorum* infection. Its expression was strongly repressed by elicitors (OGs and flg22) and fungal infection, and induced by the hormone, abscisic acid (ABA) [19]. LYK4 binds to chitin or chitooctaose, and the binding was partially repressed in a *lyk4* mutant [20]. LYK5 recognizes long-chain chitooligosaccharides and forms a complex with CERK1. This complex activates the CERK1 kinase domain and induces downstream immune responses [21]. LysM-containing glycosylphosphatidylinositol-anchored protein 2 (LYM2) is an OsCEBiP homologue in *Arabidopsis*, but the *lym2* mutant did not affect CERK1-mediated chitin responses. Instead, LYM2 participated in the CERK1-independent pathway by mediating a reduction in molecular flux in the presence of chitin [22], as well as contributed to disease resistance against *A. brassicicola* through the perception of chitin [23]. Interestingly, LYK proteins in legumes are essential receptors for the perception of lipochitooligosaccharide nodulation factors (NFs) produced by rhizobia and are essential for the establishment of nitrogen-fixing symbiosis [24–29].

Functional analysis of LysM-containing proteins demonstrated the importance of the LysM domain in fungal pathogen recognition. Although in-depth investigations have been performed in rice and *Arabidopsis*, limited information has been reported on LysM-containing proteins in apple or other woody plants. Thus, it remains unclear whether apple utilizes the same mechanism as *Arabidopsis* or rice to recognize fungal pathogens and trigger downstream defense responses. Recently, Zhou et al. [30] reported an apple CERK1 like protein, MdCERK1, which was induced by chitin and *Rhizoctonia solani*. These findings provided evidences that MdCERK1 may also play a role in apple defense responses against fungal pathogens. In this study, a gene encoding the LysM-domain containing protein was identified in apple tissues infected with *Botryosphaeria dothidea*. The corresponding protein shared highly similarity with MdCERK1 (designated as *MdCERK1–2*). Results revealed that *MdCERK1–2* overexpression (OE) in *Nicotiana benthamiana* (*Nb*) plants improved their resistance to fungal pathogens.

## Results

### Characterization of a LysM-containing protein kinase from *Malus × domestica*

**Fig. 1:**
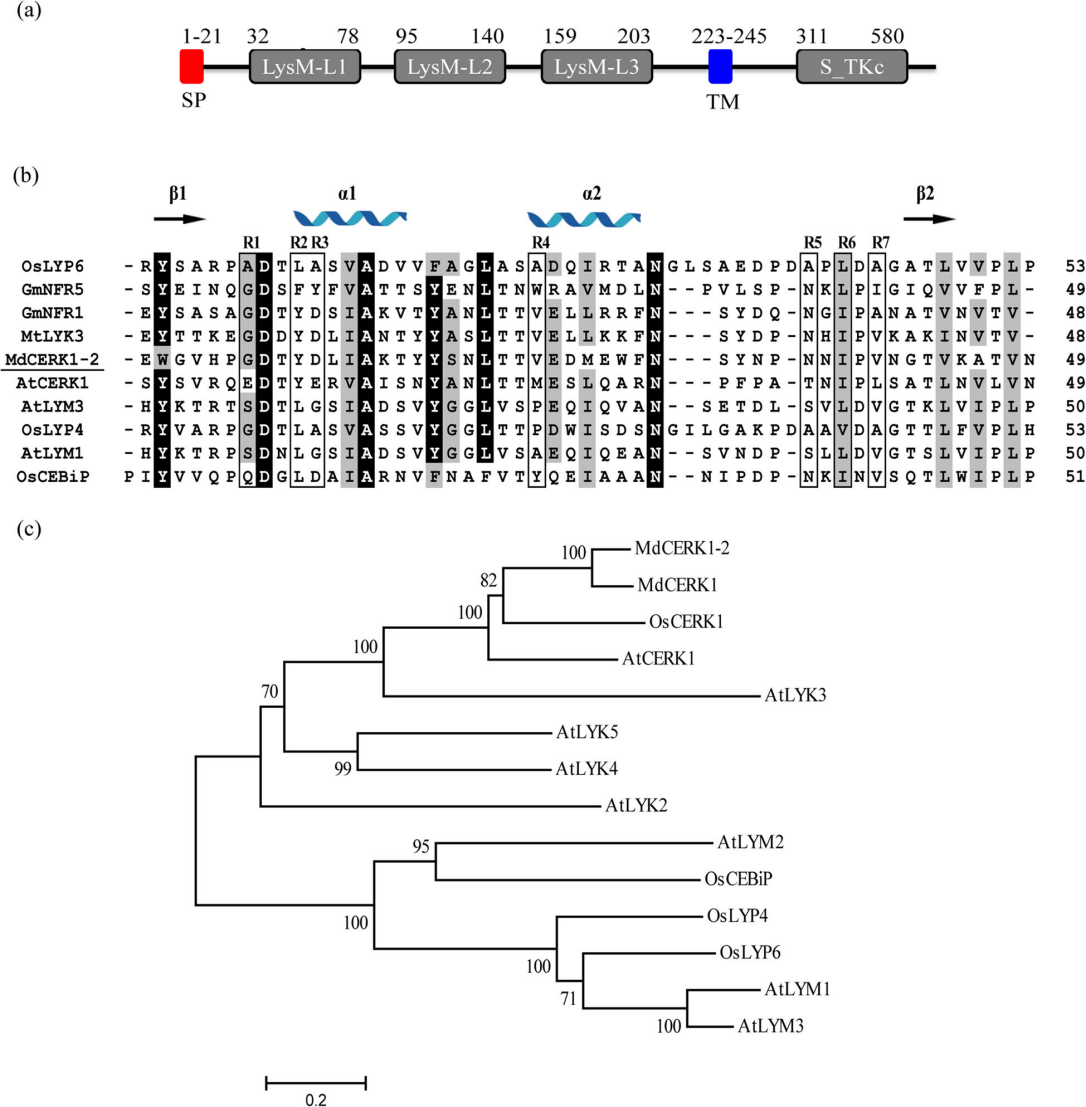
Characterization of the MdCERK1–2 protein. **a** Motifs and domains of the MdCERK1–2 protein. Numbers indicate the position of amino acids. SP, signal peptide; TM, transmembrane region. **b** Sequence alignment of LysM proteins. Consensus and similar amino acid residues for all sequences are highlighted with black or gray background. R1–R7 indicate 7 crucial positions of NAG recognition. R4, R6 and R7 are hydrophobic amino acid residues. **c** Phylogenetic tree of LYK proteins from apple, rice and Arabidopsis. The tree was constructed in MEGA 5.0 using the neighbor-joining (NJ) method. Bootstrap support values are indicated with numbers at the nodes. Proteins used in the phylogenetic tree are summarized in Table S1

A gene (GDR ID: MD17G1102100) encoding LysM-containing protein kinase was found to be highly expressed in shoot barks of apple by *B. dothidea* as revealed by transcriptome approach. Its protein contains a long extracellular region, a transmembrane domain and an intracellular Ser/Thr kinase domain, and was highly homologous to chitin recognition proteins MdCERK1 [30] and AtCERK1 [9, 10]. Furthermore, the motif analysis revealed that the extracellular region consists of a signal peptide consisting of 21 amino acids and 3 LysMs (Figs. 1a; S1). The domain composition of the newly identified protein was similar to MdCERK1. To distinguish it from MdCERK1, the newly identified gene was designated as *MdCERK1–2*. Homologous alignment revealed that the MdCERK1–2 protein shared high sequence identity with MdCERK1 (82.9%), OsCERK1 (57.6%), and AtCERK1 (57.1%) (Fig. S2). By aligning MdCERK1–2 with well-studied LysM-containing proteins, 7 residues that are crucial for NAG binding were found in LysM2, which are similar to the other reported LysM-containing proteins [14, 31] (Fig. 1b), suggesting that MdCERK1–2 can bind to NAG. The phylogenetic analysis revealed that MdCERK1–2 was closely related to MdCERK1, OsCERK1, AtCERK1, and AtLYK3 (Fig. 1c). According to early reports, these proteins are all involved in the defense against fungal pathogens [9, 19, 30], suggesting that MdCERK1–2 may also be involved in the defense responses against fungal pathogen infection.

### *MdCERK1–2* exhibited similar subcellular location and chitin-binding activity as MdCERK1 and AtCERK1

**Fig. 2:**
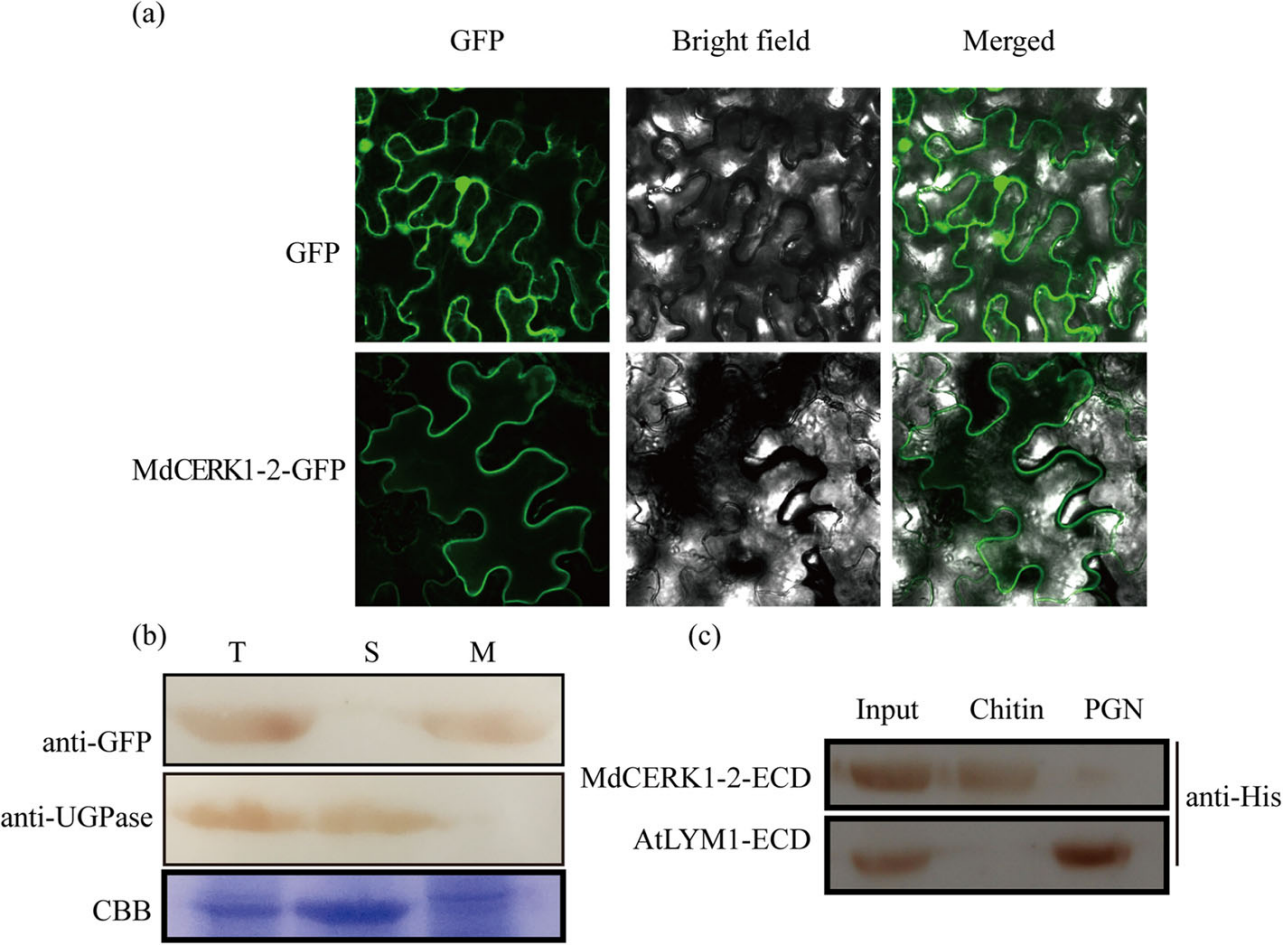
Subcellular localization and ligand binding of the MdCERK1–2 protein. **a** Subcellular localization of *MdCERK1–2*. The *MdCERK1–2*-*GFP* fusion gene was transiently expressed in *Nb* leaves using the *A. tumefaciens*-mediated method and observed with a confocal microscope (bottom). The control expressing GFP was also observed (top). **b** Membrane proteins from *Nb* plants expressing *MdCERK1–2*-GFP were prepared and separated with SDS-PAGE. The presence of MdCERK1–2-GFP in membrane proteins was determined by immunobloting with an anti-GFP antibody. S, soluble protein; T, total protein; M, membrane protein; CBB, Coomassie brilliant blue staining. Anti-UGPase was used as internal reference of cytoplasm protein. **c** MdCERK1–2-ECD binds to chitin, but does not bind to PGN. Binding proteins were separated by SDS-PAGE and detected by immunobloting with an anti-His antibody

Subcellular location revealed that MdCERK1–2 was located at the plasma membrane. Fluorescence of the MdCERK1–2-GFP fusion protein was detected at the plasma membrane (Fig. 2a). This result was confirmed by subsequent immunobloting experiments using an anti-GFP antibody. Abundant MdCERK1–2-GFP proteins existed in the microsomal fraction. No detectable MdCERK1–2-GFP was observed in the soluble fraction (Fig. 2b). Binding assays using recombinant proteins demonstrated that the putative ectodomain of MdCERK1–2 specifically bound to chitin, but did not bind to PGN (Fig. 2c). In contrast, under the same conditions, obvious binding to PGN was observed in AtLYM1, a plasma membrane protein of *Arabidopsis* that physically interacts with PGNs and mediates *Arabidopsis* sensitivity to PGNs in gram-negative and gram-positive bacteria [4].

### *MdCERK1–2* expression increased during *B. dothidea* infection

**Fig. 3:**
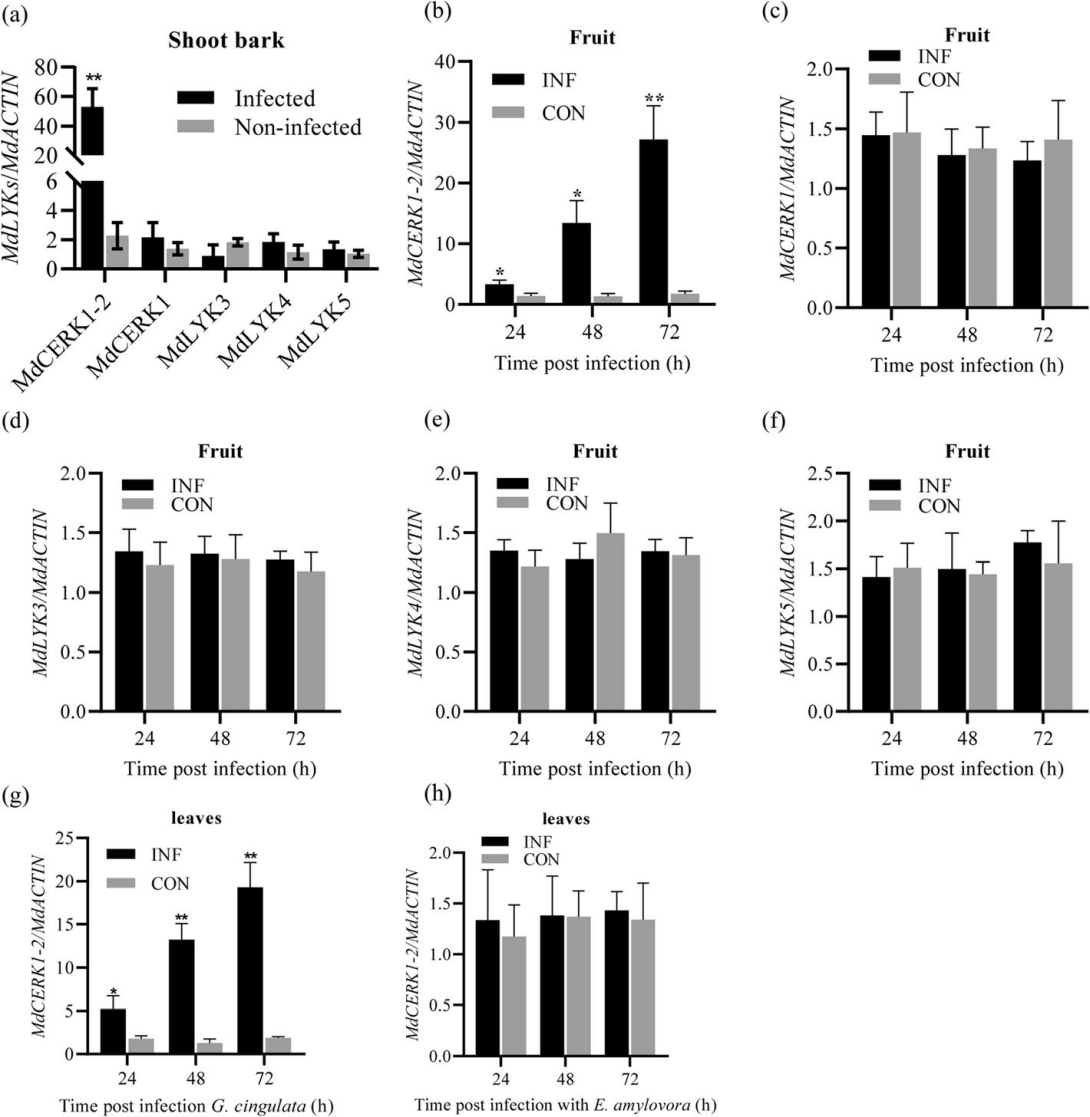
Expression of apple *MdLYKs*. **a***MdLYKs* expression in shoot bark was induced by *B. dothidea*. Samples were collected 28 dpi when warts were just visible. **b**–**f***MdLYKs* expression in fruits induced by *B. dothidea*. **g**, **h***MdCERK1–2* expression in leaves inoculated with *E. amylovora* or *G. cingulata*. INF: infected sample; CON: mock infected sample. Four replicates were used for each experiment and experiments were performed at least 3 times. The data are presented as the mean ± SD (*n* = 4) and were subjected to a two-way ANOVA followed by Tukey’s post-hoc test. Significant differences were determined when *p <* 0.05. Asterisks indicate significant differences when compared to the control (** *p <* 0.01)

To examine the expression changes in response to *B. dothidea* infection, the expressions of *MdCERK1–2* and other 4 apple *LYK* genes were analyzed, including *MdCERK1* and 3 putative *LYK* genes, *MdLYK3*, *MdLYK4*, and *MdLYK5* (Table S1). In apple shoot bark and fruits, two target tissues of *B. dothidea* infection, only *MdCERK1–2* was significantly (*p* < 0.01, *n* = 4) upregulated after *B. dothidea* infection compared to the mock-infected control (Fig. 3a, b). No significant changes were observed in the expressions of other *LYK* genes, including *MdCERK1* (Fig. 3a, c–f)*.* Next, whether *MdCERK1–2* could be induced by other apple fungal and bacterial pathogens was examined. *MdCERK1–2* expression also increased in the young leaves of apple plantlets after inoculation with the apple fungal pathogen, *Glomerella cingulate* (Fig. 3g). However, no significant expression changes of *MdCERK1–2* were observed after *Erwinia amylovora* infection*,* a bacterial pathogen (Fig. 3h). These results suggest that *MdCERK1–2* was involved in the immune defense responses against fungal pathogens.

### *MdCERK1–2* OE improved the resistance of *Nb* plants to *A. alternata*

**Fig. 4:**
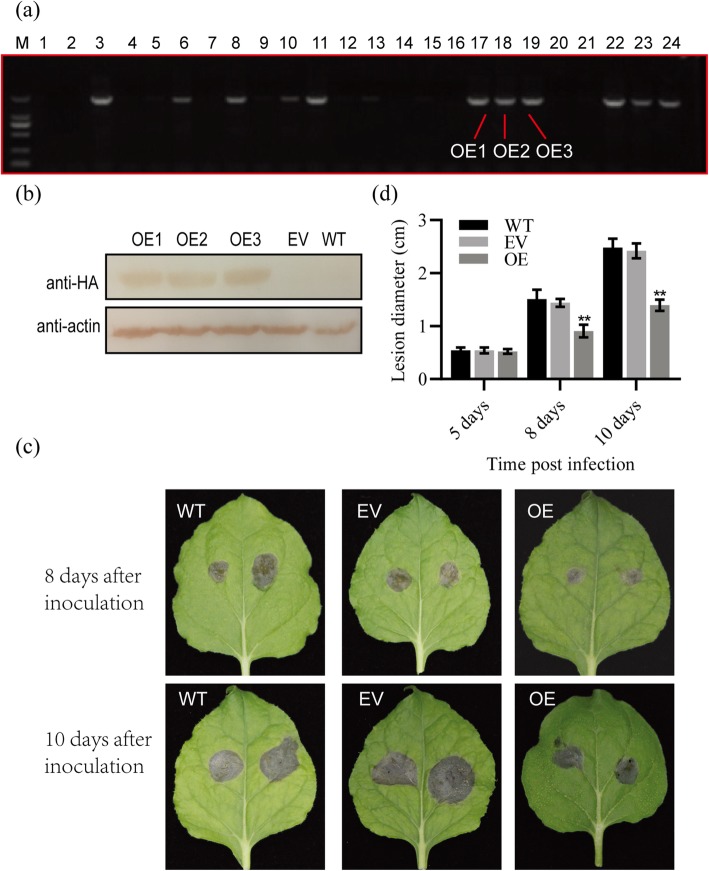
*MdCERK1–2* OE improved the resistance of *Nb* plants to *A. alternata*. **a** Transgenic plants were screened using qRT-PCR. Numbers represent different lines. **b** Positive transgenic plants were verified using immunobloting with an anti-HA antibody. **c** Representative leaves with lesions resulted from inoculation with fungal discs of *A. alternata* (0.5 cm in diameter)*.* Photos were taken at 8 and 10 dpi. **d** Quantitation of the lesions was conducted by measuring lesion diameter. The data were analyzed for statistical differences by a two-way ANOVA followed by Tukey’s post-hoc test. Bars indicate the mean ± SD (*n* = 5). Asterisks indicate significant differences when compared to the WT (** *p* < 0.01)

**Fig. 5:**
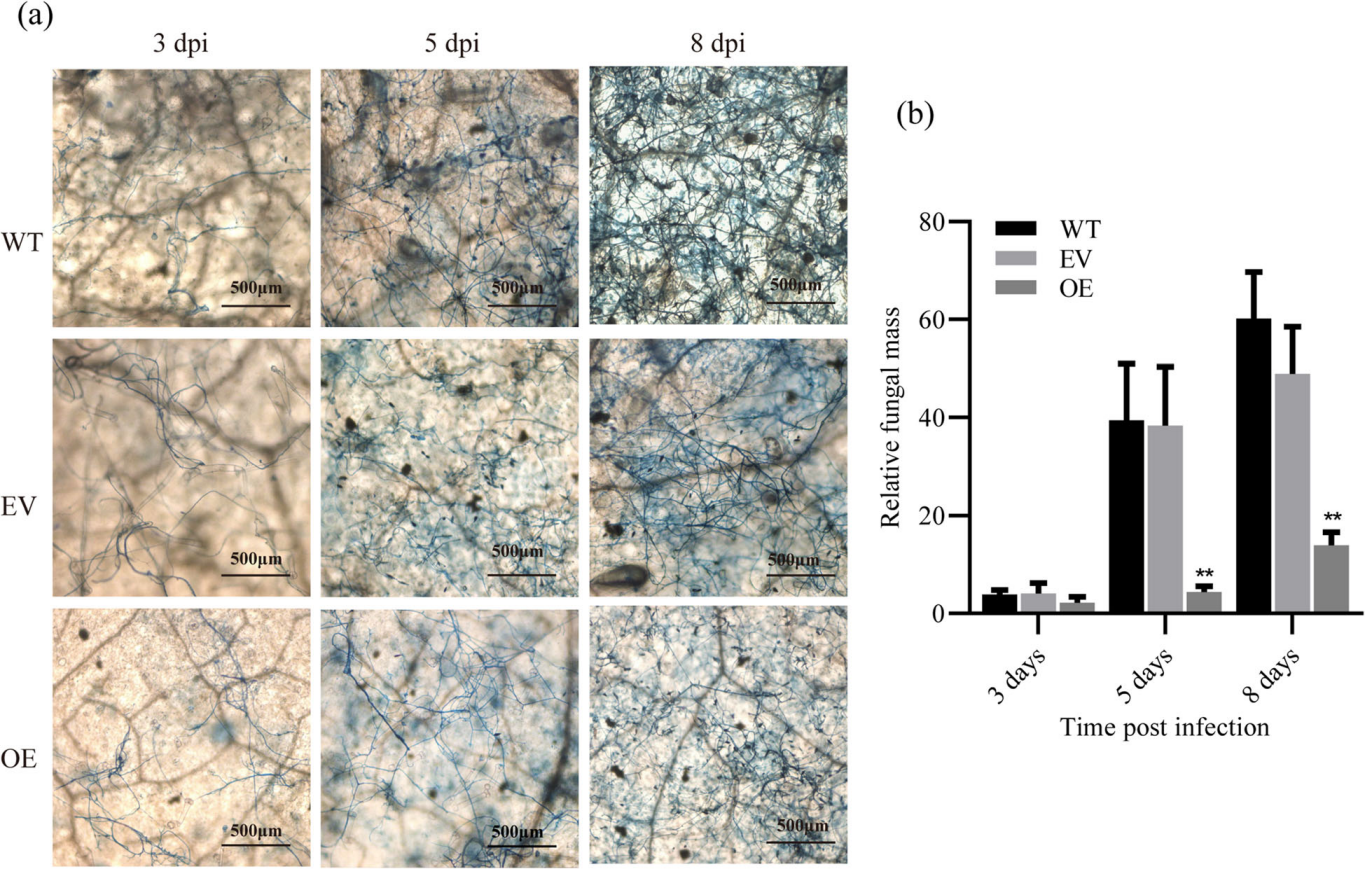
Pathogenic fungus growth on *Nb* leaves. **a** Microscopic observation of fungal hyphae. **b** Relative fungal mass as revealed by qRT-PCR. The data were analyzed for statistical differences by a two-way ANOVA followed by Tukey’s post-hoc test. Bars indicate the mean ± SD (n = 4). Asterisks indicate significant differences when compared to the WT (** *p* < 0.01)

To generate OE transgenic *Nb* plants, binary vectors carrying *MdCERK1–2* and the 3 HA tags were introduced into *Nb* using *Agrobacterium*-mediated transformation. Transgenic lines were screened using hygromycin and carbeniclllin. All of the obtained transgenic lines were confirmed by PCR with genomic DNA as template (Fig. 4a). The expression of *MdCERK1–2* was confirmed using immunobloting. The identified transgenic lines exhibited high levels of *MdCERK1–2* expression (Fig. 4b). No visible difference in phenotype was observed between transgenic and wild-type (WT) plants. Next, whether *MdCERK1–2* OE altered the resistance of *Nb* plants to pathogenic fungi was tested. *MdCERK1–2* OE plants were used for *A. alternata* inoculation. EV-transformed *Nb* and WT plants were used as the controls. No obvious lesions were observed 5 days post-inoculation (dpi)*.* Visible lesions were observed at 8 dpi on almost all OE, EV, and WT plants. However, there were significantly (*p* < 0.01, *n* = 5) milder lesions on OE plants compared to EV and WT plants. Only small lesions were observed on OE plants, while more severe lesions were observed on EV and WT plants (Fig. 4c, d). Fungal growth was also evaluated by microscopic observation and relative fungal mass was calculated. Hypha growth was observed at 3 and 5 dpi, although no visible lesions were observed. At 8 dpi, heavy hyphae growth was observed in WT and EV plants, while the relative fungal mass of OE plants was significantly (*p* < 0.01, *n* = 5) lower compared to WT and EV plants (Fig. 5a, b).

### ROS accumulation and callose deposition increased in *MdCERK1–2* OE plants

**Fig. 6:**
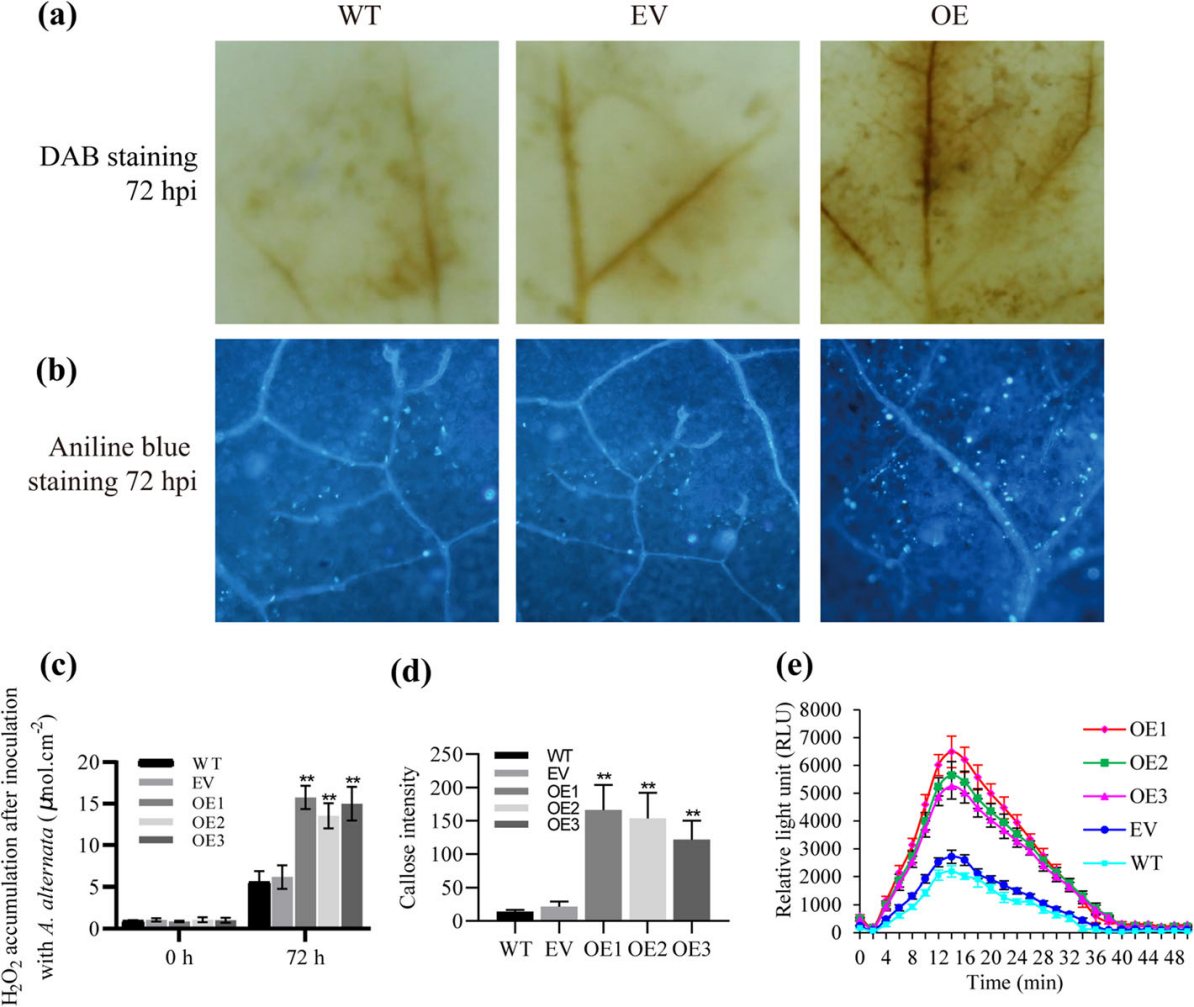
ROS accumulation and callose deposition in *Nb* leaves responding to *A. alternata* infection. **a** ROS accumulation was visualized by DAB staining and (**c**) quantified by xylenol orange assay. **b** Callose deposition was visualized by aniline blue staining and (**d**) quantified by dot counting using Image J software based on 9 photographs. **e** H_2_O_2_ generation in response to chitin treatment using luminol-based assays. The data were analyzed for statistical differences by a two-way ANOVA followed by Tukey’s post-hoc test. Bars indicate the mean ± SD of 5 independent biological replicates for H_2_O_2_ assays and 9 replicates for callose assays. Asterisks indicate significant differences when compared to the WT at the indicated time points (** *p* < 0.01)

To further examine the effects of *MdCERK1–2* on defense responses, ROS accumulation and callose deposition were evaluated using DAB and aniline blue staining, respectively. Significant (*p* < 0.01; n = 5) increases in ROS accumulation were detected in OE plants compared to EV plants at 72 h post-inoculation (hpi) with *A. alternata* (Fig. 6a). Xylenol orange assays were used to quantify ROS accumulation and confirmed the enhanced ROS levels in OE plants (Fig. 6c). No significant differences were observed among OE, EV, and WT plants under mock-infection conditions. Chitin treatment also induced significantly (*p* < 0.01; *n* = 7) higher H_2_O_2_ generation in OE plants compared to WT and EV plants (Fig. 6e). Callose deposition was visualized by aniline blue staining and quantified by digital count measurements. *MdCERK1–2* OE significantly (*p* < 0.01; *n* = 9) increased callose deposition in OE plants compared to EV and WT plants (Fig. 6b, d). These results suggest that *MdCERK1–2* OE positively regulates ROS accumulation and callose deposition in response to *A. alternata* infection.

### *MdCERK1–2* OE affects the expression of defense-related genes

**Fig. 7:**
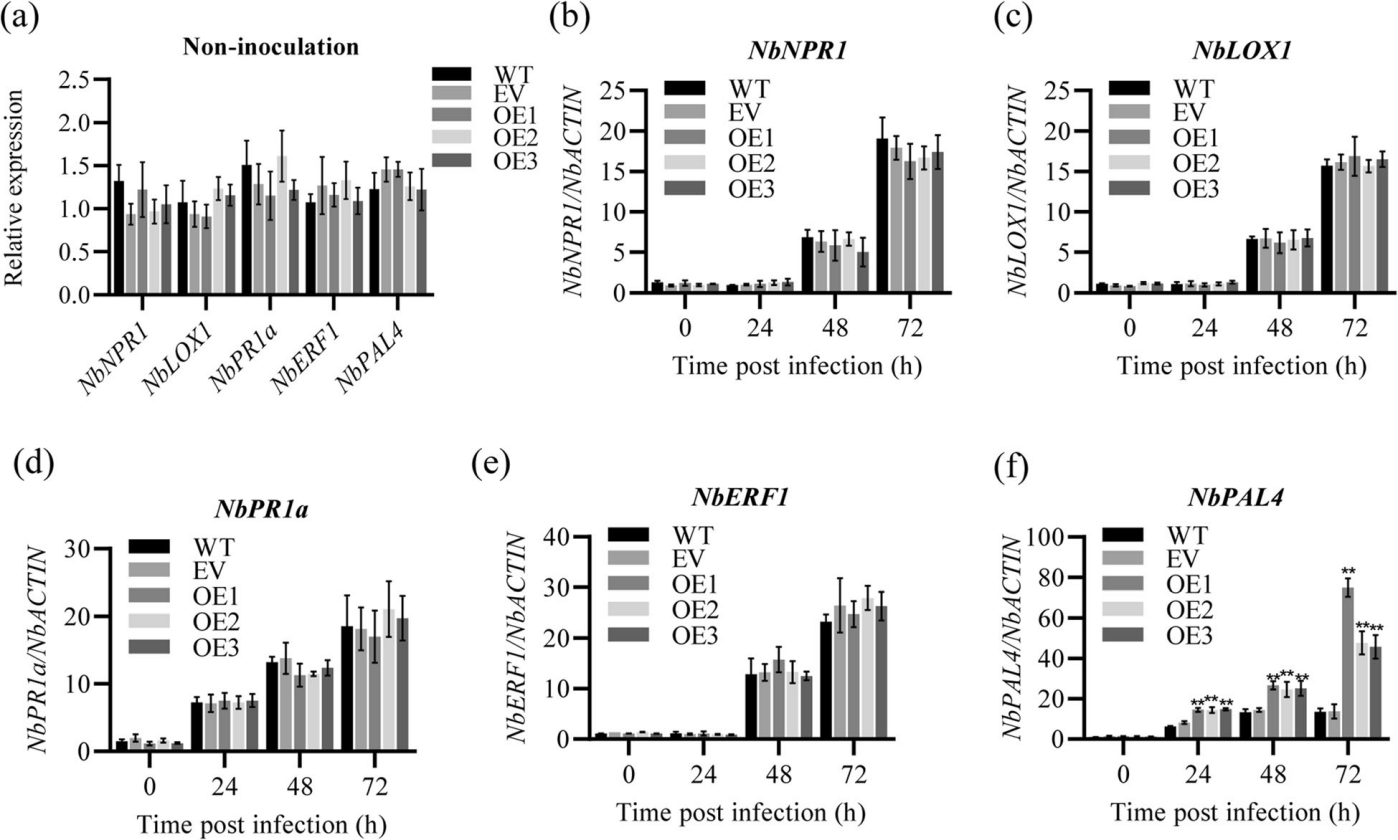
The effect of *MdCERK1–2* OE on defense-related genes in *Nb* plants. **a**, Basal expressions of defense-related genes in *Nb* plants. **b**–**f**, Induced expressions of defense-related genes by *A. alternata.* Leaves were collected at 0, 24, 48, and 72 hpi*.* The expression levels of defense-related genes were determined using qRT-PCR. Three replicates were used for each gene and 3 independent experiments were performed. The data were analyzed for statistical differences by a two-way ANOVA followed by Tukey’s post-hoc test. Asterisks indicate significant difference when compared to the WT at the indicated time points (** *p* < 0.01)

To determine whether *MdCERK1–2* OE affects the expression of defense-related genes during pathogen infection, defense-related genes were quantified by qRT-PCR at different time points after *A. alternata* inoculation (Fig. 7), including the salicylic acid (SA)-related genes, *NbNPR1* and *NbPR1a* [32, 33], jasmonic acid (JA)-responsive gene, *NbLOX1* [32, 33], ethylene-responsive gene, *NbERF1* [32], and *NbPAL4*, a gene involved in the biosynthesis of polyphenol compounds [34, 35]. The basal expression of these genes did not show significant differences among OE, EV, and WT plants (Fig. 7a). After *A. alternate* infection*, NbPAL4* exhibited significantly (*p* < 0.01, *n* = 4) higher expression in OE plants compared to EV and WT plants (Fig. 7f)*.* In contrast, *NbNPR1*, *NbPR1a*, *NbERF1*, and *NbLOX1* were not significantly different between OE and WT plants, although these genes were induced by *A. alternata* infection.

### *MdCERK1–2* OE alters polyphenolic metabolism in *Nb* plants

**Fig. 8:**
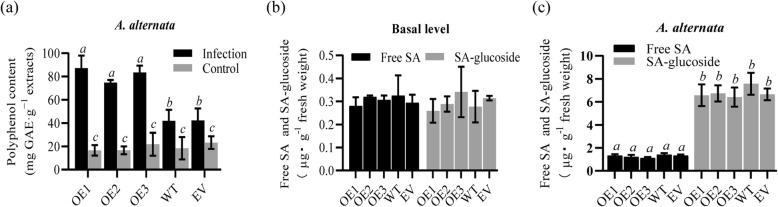
The effect of *MdCERK1–2* expression on polyphenol content and SA accumulation. **a** Polyphenol contents in OE, EV, and WT plants after *A. alternata* infection. **b** Basal SA levels in OE, EV, and WT plants. **c** SA levels in OE, EV, and WT plants after *A. alternata* infection. The data were analyzed for statistical differences by a two-way ANOVA followed by Tukey’s post-hoc test. Bars represent the mean ± SD (*n* = 4). Different letters indicate statistically significant differences; *p* < 0.05 was regarded as being statistically significant

The differences in *NbPAL4* gene expression between OE and WT plants suggest that polyphenolic metabolism and the SA signaling pathway may be influenced by *MdCERK1–2* OE. Total phenolic contents and SA levels were analyzed to determine the effects of *MdCERK1–2* OE. After *A. alternata* infection, all of the tested plants exhibited significantly enhanced polyphenol content levels (*p* < 0.05; n = 4). Polyphenol contents in OE plants were clearly higher than that in EV and WT plants (Fig. 8a). In contrast, SA levels were not significantly different (Fig. 8b, c)*. MdCERK1–2* OE did not alter basal SA levels (Fig. 8b). Although the SA levels of all of the tested plants increased after *A. alternata* infection, *MdCERK1–2* OE did not result in significant differences in SA levels between OE and WT plants or between OE and EV plants in response to *A. alternata* infection (Fig. 8c).

## Discussion

Both AtCERK1 and OsCERK1 are required for chitin signaling and plant resistance to fungal pathogens [9, 10, 12, 17, 31, 36], but their recognition mechanisms for chitin differ. It is important to determine whether the apple CERK1 homologue plays an important role in fungal pathogen defense. Data presented here indicate that the apple *MdCERK1–2* gene is also involved in the defense against fungal pathogens. Specifically, *MdCERK1–2* OE improved the resistance of *Nb* plants to *A. alternata* and affected their immune response. This work, combined with the findings reported by Zhou et al. [30], confirmed that apple CERK1 also functions as a PRR, recognizing fungal pathogen and playing an important role in apple plant defense against fungal pathogens.

The gene expression data indicate that *MdCERK1–2* was induced by 2 fungal pathogens, *B. dothidea* and *G. cingulate,* but not by the bacterial pathogen, *E. amylovora*, suggesting that its expression changes were not specifically in response to *B. dothidea.* Generally, PRRs recognize conserved microbial molecules, but not certain specific pathogens. The expression changes of *MdCERK1–2* in response to different pathogenic fungi may reflect a broad-spectrum property of MdCERK1–2 as a potential PRR. *MdCERK1–2* expression was not induced by *E. amylovora* in apple, but this was not enough to exclude its involvement in the defense against bacterial pathogens. *MdCERK1–2* may not function as a PRR in the defense against bacterial pathogens just as AtCERK1 or OsCERK1. The two proteins are required for bacteria recognition via interacting with LYM1 and LYM3 in *Arabidopsis*, or OsLYP4 and OsLYP6 in rice, which physically interact with PGN [4, 37], a bacterial PAMP.

The enhanced expression of *MdCERK1–2* after pathogenic fungi infection suggests that high *MdCERK1–2* levels are required for it to function as PRR. It is known that chitin treatment enhances *CERK1* expression and induces plant resistance to fungal and bacterial pathogens [1]. However, it has not yet been determined whether chitin-induced resistance correlates with the high levels of *CERK1*. If enhanced *CERK1* expression is required for plant resistance, there are 2 possibilities regarding *CERK1* function. One is that basal *CERK1* levels are not enough for its PRR function, thereby enhanced *CERK1* levels are necessary. The other is that *CERK1* may play additional roles that differ from that as a PRR. In previous reports, transient *CERK1* OE resulted in cell death in the absence of chitin or pathogen infection [38, 39], suggesting that *CERK1* has a function that is independent of chitin signaling or pathogen infection. In this study, *MdCERK1–2* OE improved *Nb* plant resistance to *A. alternata*, indicating that high *MdCERK1–2* levels are important for its function.

Although *MdCERK1–2* OE improved *Nb* plant resistance to fungal pathogens, no significant effects on the tested parameters were observed in the absence of chitin treatment or pathogen infection, including SA levels, phenol contents, gene expression, ROS accumulation, and callose deposition, suggesting that the function of *MdCERK1–2* in plant immune responses depends on chitin signaling or pathogen infection. Notably, *MdCERK1–2* OE resulted in increased ROS accumulation and callose deposition after *A. alternata* inoculation. This may represent a non-specific enhancement of defense responses mediated by *MdCERK1–2* against microorganism invasion. ROS accumulation and callose deposition widely contribute to plant defense responses against fungal infections and are affected by LysM protein-medicated signaling [9, 38]. Enhanced ROS accumulation and callose deposition in OE plants indicate that ROS accumulation contributed to the improved resistance of *Nb* plants to fungal pathogens.

*MdCERK1–2* OE potentiated *NbPAL4* expression in response to *A. alternata* infection. Other tested genes, including *NbNPR1, NbPR1a, NbLOX1*, and *NbERF1*, did not exhibit significant changes. These results suggest that the enhancement of resistance in OE plants to fungal pathogens compared to WT was related to polyphenolic metabolism, not to the SA, JA, or ethylene pathways. Several previous reports regarding LysM-containing protein-mediated chitin signaling found that it was independent of the SA, JA, or ethylene signaling pathways, but it was related to phytoalexin [11, 19]. The data also showed significantly enhanced total phenolic contents in OE plants after *A. alternata* infection, which supports the influence of *MdCERK1–2* on polyphenolic metabolism.

Previous studies found that CERK1 affects SA signaling pathways. Mutations in the ectodomain of CERK1 promote the accumulation of SA and enhances the resistance to biotrophic pathogens [40], while mutations in the kinase domain of CERK1 did not affect SA-induced defense responses [11]. SA regulates CERK1 levels and potentiates chitin-induced responses [41]. However, the results of this study found no significant differences in the SA levels between OE and WT plants after *A. alternata* infection, suggesting that the improvement of *MdCERK1–2*-mediated resistance did not result from the effects of *MdCERK1–2* OE on SA levels.

## Conclusions

An apple LyM-containing protein gene, *MdCERK1–2*, was identified in this study. *MdCERK1–2* was involved in the anti-fungal defense responses of apple as a PRR. *MdCERK1–2* OE improved the resistance of *Nb* plants to *A. alternata* infection*.* ROS accumulation, callose deposition, and polyphenols contributed to improved resistance.

## Methods

### Plant materials

The ‘Fuji’ apple cultivar was obtained from the LAIXI breeding farm of the fruit nursery stock located in Qingdao, China, and confirmed by Hongyi Dai and Yugang Zhang. Two-year-old ‘Fuji’ apple trees were used for gene cloning and expression analysis. Trees were grown in a greenhouse under natural daylight conditions. *Nb* seeds were obtained from the Qingzhou Tobacco Research Institute of China National Tobacco Company, and were confirmed by Qiming Chen. Transgenic and WT *Nb* plants were cultured in a plant growth chamber under a 16/8-h light/dark photoperiod at 26 °C/22 °C.

### Pathogen inoculation

For gene expression analysis in apple, current-year shoots were inoculated with *B. dothidea* as previously described [42]. Before inoculation, shoots were cleaned with 75% ethanol for surface sterilization. Mycelial strips were made from well-grown *B. dothidea* PDA plates with blades and wrapped onto the surface of shoots using polyethylene film. After 3 weeks, polyethylene film and mycelial strips were removed and the inoculated shoots were monitored for ring rot symptoms every 2 days. When visible warts had just formed, the barks of diseased shoots were collected and used for gene expression analysis. The same procedure was performed on other current-year shoots, except for substituting PDA for mycelial strips; these shoots were used as mock-inoculated controls.

For inoculation in *Nb* plants, the detached leaves from 7-week-old plants were placed on 1% agar in petri dishes. Mycelial plugs (5 mm diameter) were made from PDA plates of actively growing *A. alternata* and cultured at 25 °C for 1 week. Mycelial plugs were laid on detached *Nb* leaves for inoculation and kept at 25 °C. The fungal biomass in infected *Nb* leaves was determined at 3, 5, and 8 dpi by qRT-PCR using specific primers for the *AaACTIN* gene of *A. alternata* (Table S2), and normalized to the *NbACTIN* gene according to previously reported methods [43]. Trypan blue staining was used to detect mycelial growth and cell death according to previously described methods [44].

### Gene cloning and expression analysis

To clone full-length coding regions (CDS) of *MdCERK1–2*, primers were designed according to the sequences obtained from GDR and used for gene amplification. Total RNA were isolated using an EASYspin plant RNA rapid extraction kit (YP Biotech Co., Ltd., Beijing, China) and cDNA was synthetized using a PrimeScript™ II 1st strand cDNA synthesis kit (TaKaRa, Beijing, China) following the manufacturer’s instructions. qRT-PCR was performed as previously described [42]. The primers used in the gene cloning and expression analysis are provided in Table S2.

### Subcellular localization

Full-length CDS of *MdCERK1–2* was integrated into pCAMBIA1300–221-GFP upstream of the GFP sequence to form a fusion protein with GFP. The resultant construct was transformed into *A. tumefaciens* EHA105. Transformed bacteria were cultured and resuspended in buffer (10 mM MgCl_2_, 10 mM 2-(N-morpholine)-ethanesulfonic acid (MES)-NaOH, pH 5.6, and 150 μM acetosyringone) [38]. The bacterial concentration was adjusted to a final OD value of 0.5 at 600 nm and infiltrated into 4-week-old *Nb* leaves with a needleless syringe. Three days after infiltration, the infiltrated area was observed using a TCS SP5 confocal microscope (Leica Microsystems, Wetzlar, Germany) to localize MdCERK1–2-GFP fusion proteins. To verify the membrane localization of *MdCERK1–2*, microsomal and soluble proteins were prepared from *Nb* leaves transiently expressing MdCERK1–2-GFP following previously described methods [10]. The presence of MdCERK1–2-GFP was determined by immunobloting with an anti-GFP antibody (Abcam, Shanghai, China).

### In vitro chitin and PGN binding assays

The *MdCERK1–2* ectodomain was expressed and purified following previously described methods [45] with minor modifications. DNA fragments encoding the *MdCERK1–2* ectodomain were amplified from cDNA of apple branch bark and inserted into pET-28a (+) between *Nco*I and *Xho*I. Recombinant DNA molecules were transformed into *E. coli* BL21 (DE3) for protein expression. Recombinant proteins were purified using a Ni-NTA column (GE Healthcare, Shanghai, China) under denatured conditions and refolded using the gradient dialysis method [45]. Protein concentrations were determined using the BCA method [46]. The same procedure was performed with AtLYM1-ECD, which were used as positive controls in the PGN binding experiment.

For in vitro chitin binding assays, the recombinant protein buffer was changed to binding buffer (500 mM NaCl, 20 mM Tris-HCl, 1 mM EDTA, and 0.05% Triton X-100). The proteins were adjusted to a final concentration of 1.5 mg/mL. Chitin magnetic beads (100 μL, NEB) were washed 3 times with 1 mL binding buffer and mixed with 50 μL proteins (details outlined above) followed by incubation at 4 °C for 1 h. After centrifugation, magnetic beads were washed 3 times with binding buffer. Then, bead-binding proteins were eluted by boiling in 50 μL SDS-PAGE loading buffer and detected by running 20 μL on a 15% denaturing protein gel.

For the PGN binding assay, PGN (50 μg) was mixed with purified proteins in 250 μL 100 mM PBS (pH 7.0) and incubated at 4 °C for 10 min. After centrifugation at 12000×g for 10 min at 4 °C. The resultant pellet was washed 3 times with PBS (pH 7.0), dissolved in 100 μL SDS sample buffer, and separated with SDS-PAGE followed by immunoblot with anti-His antibodies (Abcam, Shanghai, China).

### Genetic transformation of *Nb* plants

The genetic transformation of *Nb* plants was performed using the *A. tumefaciens*-mediated leaf disc method [42]. The encoding cDNA of *MdCERK1–2* was integrated downstream of the 35S promoter of pCAMBIA1300–221-HA. The resultant construct was transferred into the *A. tumefaciens* strain, EHA105, which was subsequently used for genetic transformation. The genetic transformation of *Nb* plants and verification of positive plants were conducted as previously described [42].

### Histochemistry and ROS measurements

H_2_O_2_ accumulation and cell death were visualized by DAB and trypan blue staining, respectively, following previously described methods [47]. Briefly, leaves were incubated in a 3,3-diaminobenzidine (DAB) solution (1 mg/mL) overnight in the dark. Then, leaves were destained with a mixture of 80% ethanol and placed in a water bath at 65 °C. H_2_O_2_ quantitation was performed based on xylenol orange assays [47]. For measurements of H_2_O_2_ generation after treatment with chitin (200 μg/mL), Luminol-based assays were performed according to previously described methods [48].

### Microscopic observation and quantification of callose deposition

Callose deposition was examined according to previously described methods [49]. Briefly, *Nb* leaves inoculated with *A. alternata* were destained in a mixture of distilled water, glycerol, lactic acid, phenol saturated with water, and absolute ethanol at a ratio of 1:1:1:1:8 followed by staining with 0.01% aniline blue (w/v). Callose accumulation was examined using UV epifluoresence microscopy (350 nm/425 nm excitation/emission wavelengths, respectively) and quantified with digital photographs using Image J software. Callose measurements were determined based on 9 photographs and analyzed for statistical differences by a one-way ANOVA followed by Tukey’s post-hoc test. A significance threshold of *p* < 0.05 was used to determine significant differences.

### Measurement of total polyphenols and salicylic acid

Total polyphenolic contents were measured according to previously described methods [50] with minor modifications. Fresh leaves (4 g) were collected and homogenized in 8 mL methanol. After centrifugation, the supernatant was used for total polyphenol measurements. Gallic acid was used as a standard reference and the values of polyphenols were expressed as gallic acid equivalents in per gram fresh weight of the leaves. SA levels were determined according previously described methods [42].

## Supplementary information

**Additional file 1: Figute S1.** CDS and amino sequences of MdCERK1–2. Bold letters with gray shadow indicate signal peptides. Letters in boxes indicate LysMs. Underlined letters indicate transmembrane regions. Letters with wavy lines indicate the catalytic domain of Ser/Thr protein kinases.

**Additional file 2: Figure S2.** Multiple sequence alignment among MdCERK1–2, AtCERK1, and OsCERK1 proteins. Identical amino acids are indicated with white letters and a black background. A gray background indicates high levels of similarity. Gaps are indicated by dashes to improve the alignment.

**Additional file 3: Figure S3.** The original blot presented in Fig. 2b (Top).

**Additional file 4: Figure S4.** The original blot presented in Fig. 2b (Middle).

**Additional file 5: Figure S5.** The original blot presented in Fig. 2c (Bottom).

**Additional file 6: Figure S6.** The original blot presented in Fig. 2c (Top).

**Additional file 7: Figure S7.** The original blot presented in Fig. 2c (Bottom).

**Additional file 8: Figure S8.** The original blot presented in Fig. 4b (Top).

**Additional file 9: Figure S9.** The original blot presented in Fig. 4b (Bottom).

**Additional file 10: Table S1.** The genes used in the present study.

**Additional file 11: Table S2.** The primers used in this study.

## Data Availability

The datasets used and/or analysed during the current study available from the corresponding author on reasonable request.

## Abbreviations

ABA: Abscisic acid
CEBiP: Chitin elicitor binding protein
CERK1: Chitin elicitor receptor kinase1
DAB: 3, 3-diaminobenzidine
EFR: Elongation factor-tu receptor
elf18: EF-Tu epitope
EV: Empty vector-transformed plants
flg22: Bacterial flagellin epitope
FLS2: Flagellin sensing 2
LYK: Lysin motif-containing receptor-like kinase
LYM: LysM-containing glycosylphosphatidylinositol-anchored protein
LysM: Lysin motif
NAG: β-1, 4-linked N-acetyl-glucosamine
*Nb*: *Nicotiana benthamiana*
NF: Nodulation factor
OE: *MdCERK1–2*-overexpressing plants
PAMP: Pathogen-associated molecular pattern
PDA: Potato dextrose agar
PEPR: pep1 receptor
PGN: Peptidoglycan
PR: Pathogenesis-related protein
PRR: Pattern recognition receptor
PTI: PAMP-triggered immunity
Peps: Plant elicitor peptides
ROS: Reactive oxygen species
WT: Wild type plants
