# Supplementary material for: Overexpression of an apple LysM-containing protein gene, MdCERK1–2, confers improved resistance to the pathogenic fungus, Alternaria alternata, in Nicotiana benthamiana
Source: BMC Plant Biol. 2020 Apr 8;20:146. doi: 10.1186/s12870-020-02361-z (PMC7386173; doi:10.1186/s12870-020-02361-z)
Supplement: Supplementary file 1 — Additional file 1: Figute S1. CDS and amino sequences of MdCERK1–2. Bold letters with gray shadow indicate signal peptides. Letters in boxes indicate LysMs. Underlined letters indicate transmembrane regions. Letters with wavy lines indicate the catalytic domain of Ser/Thr protein kinases. [file 12870_2020_2361_MOESM1_ESM.docx]

**>****MdCERK1-2-CDS**

ATGGGATTTCGAATCGGGTTAGGGTTCTTGGTGCTGGTTTCCCTCTGTTTTACGGTGAAATCCCAGTGCAGCAAGAGCTGCGGCGCTCTAGCTTCTTACTACGTATGGCAGGACTCTAATCTCACTTTCATCGGCGAAGTCATGGGTACGACCGACGTCACCATCGCCAACTACAACAAGGACTTGGTTCCCAGCAAAGACAGCGTCCGATGGGGCATCAGGGTCAATGTTCCATTCACCTGCGGCTGCATCAATGGCGACTTCCAAGGCCATATGTTCGAGTGGGGCGTCCATCCCGGCGACACGTACGACCTGATTGCGAAGACATACTATTCTAATTTGACGACGGTGGAGGACATGGAGTGGTTCAATAGCTATAATCCTAATAATATACCCGTAAATGGTACGGTGAAAGCTACTGTGAATTGTACGTGTGGGAATAGCGCGATTTCGAAGAAATATGGTTTGTTTATTACTTATCCTCTGCGTCCGGAGGACAATTTGGCATCGATTGCGCAGACGGAGCAGCTGGATCAGACTTTGCTGCAGAGCTATAATCCTGGTGTGAATTTTAGCCAAGGGAGCGGTTTCGTGTATATTCCGGGCAAAGATCAAAACGGAAACTATTTGTCTTTGACGTCAAGTTCAGGACTGAAAGTTGGAGCCATTGCTGGCATATCTGTGGGAGTAATTGCTGGAGTGCTGCTATTGGCTGGTGGTGTATATTTTGGTTTTTTCCGGAAGAACAAGGTGGATACGAATTTGCTCCTAGCAAGATCTGAAGATCAATCTTCTCAAAATGGGCGTCCTCTTGGGATTACCCCTGATAAACCTGAAGAGTCAAATGCTGCTGGTCGAGGCCTAACAGGCATTTCTGTGGACAAATCAGTGGAGTTCTCATATGAAGAACTTGCTAGGGCTACTGATAACTTCAGCCTGGCTAATAAGATTGGACAAGGAGGTTTCGGGGCTGTTTACTATGCAGAATTGAGAGGCGAGAAAGCTGCAATCAAGAAGATGGATATGCAAGCATCAAAAGAATTTTTGGCCGAGTTAAATGTTTTGACACGTGTCCATCACTTGAATCTGGTGCGCTTGATTGGTTATTGTGTTGAAGGCTCTCTTTTCCTAGTCTATGAATACATTGAGAATGGAAACTTAAGCCAACATCTGCGCGGTTCAGGGAGGGACCCGCTACCGTGGTCTAATAGAGTGCAGATTGCCCTAGATTCAGCAAGAGGTCTGGAATACATTCATGAACATACTGTTCCTGTTTATATCCACCGCGATATCAAATCAGCCAATATATTGATAGACAAGAATTCCCATGCCAAGGTTGCAGATTTTGGGTTAACTAAACTGACTGAAGTTGGAAGTACATCACTCCCCACACGTCTTGTGGGAACATTTGGATATATGCCACCGGAATATGCTCAATATGGCGAAGTTTCTCCCAAAGTAGATGTTTATGCTTTTGGAGTTGTCATTTATGAACTGATCTCTGCCAAAGAAGCTGTTGTAAAAGCGGATGGCTCGAGTTCCGAATCAAGAGGCCTTGTTGGTTTGTTTGAAGAGGTCCTTAATCAGCCTGATGCCGAAGACCTTCGAAAATTGGTTGACCCTAACCTTGGAGACAACTATCCGCTTGATTCAGTGCGCAAGATGGCCCAACTCGCCAAAGCATGCACGCACGAAAACAAAGATCTCCGTCCAAGTATGCGATCGATCGTGGTGGCGTTGATGACACTTTCATCCTCAACTGAGGATTGGGATGTCGGATCTTTCTATGAAAATCAAGCTCTTGTCAATCTGATGTCCGGAAGGTAG

**>** **MdCERK1-2 protein**

**MGFRIGLGFLVLVSLCFTVKS**QCSKSCGALASYYVWQDSNLTFIGEVMGTTDVTIANYNKDLVPSKDSVRWGIRVNVPFTCGCINGDFQGHMFEWGVHPGDTYDLIAKTYYSNLTTVEDMEWFNSYNPNNIPVNGTVKATVNCTCGNSAISKKYGLFITYPLRPEDNLASIAQTEQLDQTLLQSYNPGVNFSQGSGFVYIPGKDQNGNYLSLTSSSGLKVGAIAGISVGVIAGVLLLAGGVYFGFFRKNKVDTNLLLARSEDQSSQNGRPLGITPDKPEESNAAGRGLTGISVDKSVEFSYEELARATDNFSLANKIGQGGFGAVYYAELRGEKAAIKKMDMQASKEFLAELNVLTRVHHLNLVRLIGYCVEGSLFLVYEYIENGNLSQHLRGSGRDPLPWSNRVQIALDSARGLEYIHEHTVPVYIHRDIKSANILIDKNSHAKVADFGLTKLTEVGSTSLPTRLVGTFGYMPPEYAQYGEVSPKVDVYAFGVVIYELISAKEAVVKADGSSSESRGLVGLFEEVLNQPDAEDLRKLVDPNLGDNYPLDSVRKMAQLAKACTHENKDLRPSMRSIVVALMTLSSSTEDWDVGSFYENQALVNLMSGR

Figure S1. Nucleotide sequence of *MdCERK1-2* gene and amino acid sequence of MdCERK1-2 protein. Letters with black box indicate Lys Motifs and letters underlined indicate transmembrane region. Bold black letters indicate signal peptide.
