# Supplementary material for: Overexpression of an apple LysM-containing protein gene, MdCERK1–2, confers improved resistance to the pathogenic fungus, Alternaria alternata, in Nicotiana benthamiana
Source: BMC Plant Biol. 2020 Apr 8;20:146. doi: 10.1186/s12870-020-02361-z (PMC7386173; doi:10.1186/s12870-020-02361-z)
Supplement: Supplementary file 2 — Additional file 2: Figure S2. Multiple sequence alignment among MdCERK1–2, AtCERK1, and OsCERK1 proteins. Identical amino acids are indicated with white letters and a black background. A gray background indicates high levels of similarity. Gaps are indicated by dashes to improve the alignment. [file 12870_2020_2361_MOESM2_ESM.pdf]

```
MdCERK1    MGFRIGLG--FLVLVSLCF-----TVKSQCSKSCG-ALASYYVWQDSNLTFIGEVMGTTD   52
MdCERK1-2  MGFRIGLG--FLVLVSLCC-----TVKPQCSKSCD-ALASYYVWQGTNLSFIGDIMNTTD   52
AtCERK1    MKLKISLIAPILLLFSFFF-----AVESKCRTSCPLALASYYLENGTTLSVINQNLNSSI   55
OsCERK1    MEASTSLLVLVLAAAAFAAGTVTEAAGDGCSAGCDLALASFYVTPNQNVTNMADLFGIGA   60

MdCERK1    VTIANYNKD-------LVPSKDSVRWGIRVNVPFTCGCI------NGDFQGHMFEWGVHP   99
MdCERK1-2  ATIVTYNKD-------SVPNIYSVRFGIRVNVPFTCGCI------NSDFHGQMFEWDVRT   99
AtCERK1    APYDQINFDPILRYNSNIKDKDRIQMGSRVLVPFPCECQ------PGDFLGHNFSYSVRQ   109
OsCERK1    ANYRSLAPYN-----PNIPNLDFINVGGRVNVYFTCGCRSLPGSPGATYLAGAFPFQMSR   115

MdCERK1    GDTYDLIAKTYYSNLTTVEDMEWFNSYNPNNIPVNGTVKATVNCTCGNSAISKKYGLFIT   159
MdCERK1-2  GDTYDQIAKIFYSNLTTVEDLEWFNSYDPNNILNNTKVNVTVNCTCGNSTVSEDYGLFIT   159
AtCERK1    EDTYERVAISNYANLTTMESLQARNPFPATNIPLSATLNVLVNCSCGDESVSKDFGLFVT   169
OsCERK1    GQIYTSVAAN-YNNLTTAEWLQATNSYPANNIPDTAVINATVNCSCGDASISPDYGLFLT   174

MdCERK1    YPLRPEDNLASIAQTEQLD--QTLLQSYNPGVN----FSQGSGFVYIPGKDQNGNYLSLT   213
MdCERK1-2  YPLRPEDSLDSIAETEQLD--QALLQRYNPGVFEEALFSILPTKVSFYLADQNGTYLPLK   217
AtCERK1    YPLRPEDSLSSIARSSGVS--ADILQRYNPGVN----FNSGNGIVYVPGRDPNGAFPPFK   223
OsCERK1    YPLRAEDTLASVAATYGLSSQLDVVRRYNPGMES----ATGSGIVYIPVKDPNGSYLPLK   230

MdCERK1    SSSG---------------LKVGAIAGISVGVIAGVLLLAGG-VYFGFFRKNKVDTNLLL   257
MdCERK1-2  SSSGDTAVSKIGFMFRCQWLGGGAIGGISVGVIAGVLLLAGGGIYFGIFRKNKVDTKFLL   277
AtCERK1    SSKQDG-------------VGAGVIAGIVIGVIVALLLILFI-VYYAYRKNKSKGDSFSS   269
OsCERK1    SPGKG--------------ASAGAIAG---GVVAGVVVLAAIFLYIIFYRRRKAKQATLL   273

MdCERK1    ARSEDQSSQNGRPLGITPDKPEESNAAGRGLTGISVDKSVEFSYEELARATDNFSLANKI   317
MdCERK1-2  ATSEDQSSPNGRSLVITPDKPGESNAAGRGRTGISVDKSVEFSYEELARATDNFSLANKI   337
AtCERK1    SIPLSTKADHASSTSLQSGGLGGAGVS-PGIAAISVDKSVEFSLEELAKATDNFNLSFKI   328
OsCERK1    QSSED--STQLGTISMDKVTP-STIVGPSPVAGITVDKSVEFSYEELSNATQGFSIGNKI   330

MdCERK1    GQGGFGAVYYAELRGEKAAIKKMDMQASKEFLAELNVLTRVHHLNLVRLIGYCVEGSLFL   377
MdCERK1-2  GQGGFGAVYYAELRGEKAAIKKMDMQASKEFLAELNVLTRVHHLNLVRLIGYCVEGSLFL   397
AtCERK1    GQGGFGAVYYAELRGEKAAIKKMDMEASKQFLAELKVLTRVHHVNLVRLIGYCVEGSLFL   388
OsCERK1    GQGGFGAVYYAELRGEKAAIKKMDMQATHEFLAELKVLTHVHHLNLVRLIGYCIESSLFL   390

MdCERK1    VYEYIENGNLSQHLRGSGR--DPLPWSNRVQIALDSARGLEYIHEHTVPVYIHRDIKSAN   435
MdCERK1-2  VYEYIENGNLSQHLRGSGRGRDPLPWSIRVQIALDSARGLEYIHEHTVPVYIHHDIKSAN   457
AtCERK1    VYEYVENGNLGQHLHGSGR--EPLPWTKRVQIALDSARGLEYIHEHTVPVYVHRDIKSAN   446
OsCERK1    VYEFIENGNLSQHLRGMGY--EPLSWAARIQIALDSARGLEYIHEHTVPVYIHRDIKSAN   448

MdCERK1    ILIDKNSHAKVADFGLTKLTEVGSTSLPTR--LVGTFGYMPPEYAQYGEVSPKVDVYAFG   493
MdCERK1-2  ILIDKNFHAKVADFGLTKLTEVGSTSLPTR--LVGTFGYMPPEYAQYGEVSPKIDVFAFG   515
AtCERK1    ILIDQKFRAKVADFGLTKLTEVGG-SATRG--AMGTFGYMAPE-TVYGEVSAKVDVYAFG   502
OsCERK1    ILIDKNYRAKVADFGLTKLTEVGGTSMPTGTRVVGTFGYMPPEYARYGDVSPKVDVYAFG   508

MdCERK1    VVIYELISAKEAVVKADGSSSESRGLVGLFEEVLNQPD-AEDLRKLVDPNLGDNYPLDSV   552
MdCERK1-2  VVIFELISAKEAVFREDGSSSESKGLVGLFEEVLNQPD-LEDLRKLVDPNLGDNYPLDSL   574
AtCERK1    VVLYELISAKGAVVKMTEAVGEFRGLVGVFEESFKETDKEEALRKIIDPRLGDSYPFDSV   562
OsCERK1    VVLYELISAKEAIVRSTESSSDSKGLVYLFEEALNSPDPKEGLRTLIDPKLGEDYPIDSI   568

MdCERK1    RKMAQLAKACTHENKDLRPSMRSIVVALMTLSSSTEDWDVGSFYENQALVNLMSGR       608
MdCERK1-2  HKMAQLGKACTHENKDLRPSMRSIVVALMTLSSSTEDWDVRSFYENQALVNLMSGR       630
AtCERK1    YKMAELGKACTQENAQLRPSMRYIVVALSTLFSSTGNWDVG-NFQNEDLVSLMSGR       617
OsCERK1    LKLTQLAKVCTQEDPKLRPSMRSVVVALMTLSSTSEFWDMNNLYENQGLVNLMSGR       624
```

Figure S2. Multiple sequence alignment among MdCERK1-2, AtCERK1, and OsCERK1 proteins. Identical amino acids are indicated with white letters and a black background. A gray background indicates high levels of similarity. Gaps are indicated by dashes to improve the alignment.
