# Supplementary figures and images for: Overexpression of an apple LysM-containing protein gene, MdCERK1–2, confers improved resistance to the pathogenic fungus, Alternaria alternata, in Nicotiana benthamiana
Source: BMC Plant Biol. 2020 Apr 8;20:146. doi: 10.1186/s12870-020-02361-z (PMC7386173; doi:10.1186/s12870-020-02361-z)

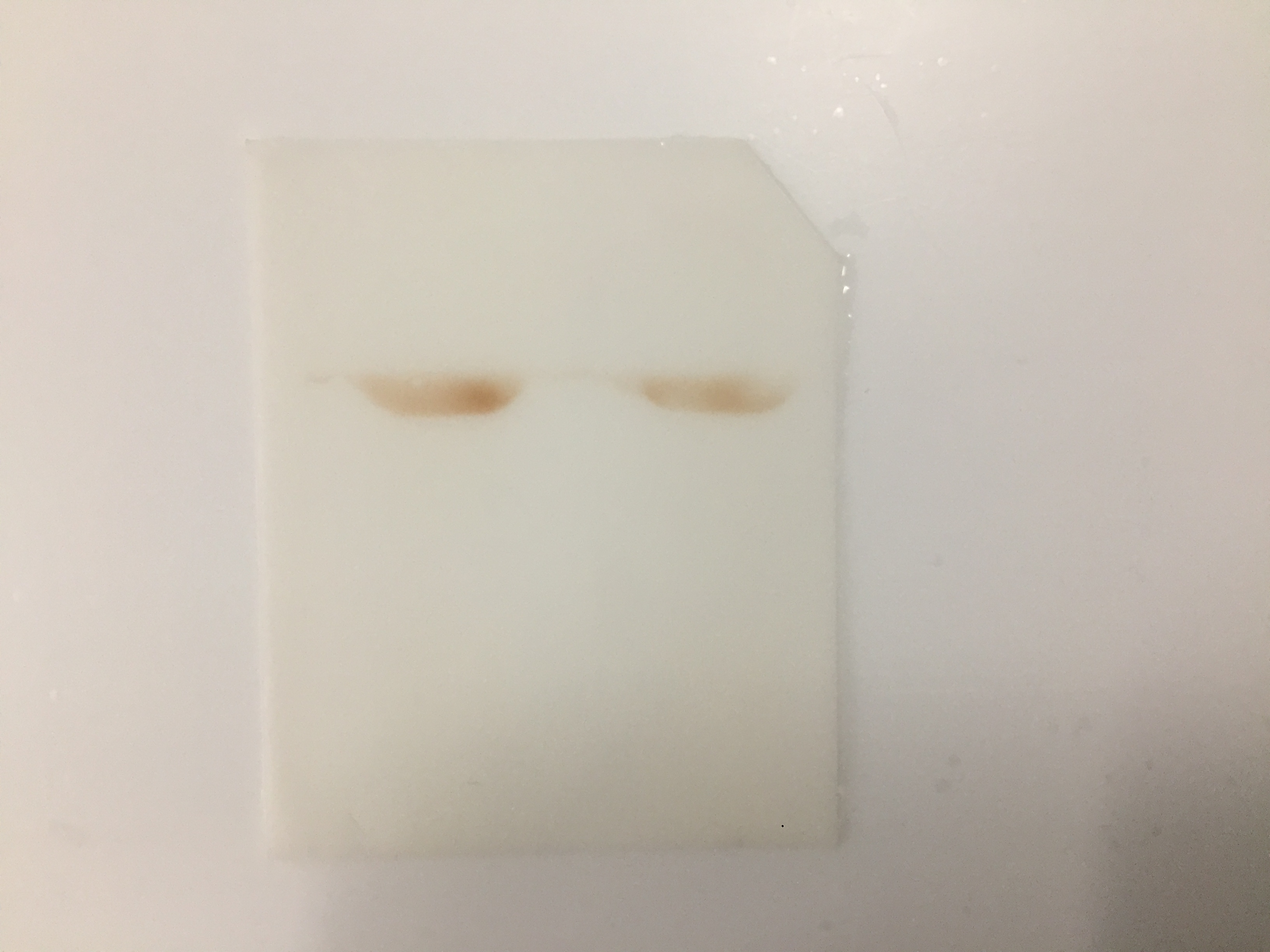

Supplement: Supplementary file 3 — Additional file 3: Figure S3. The original blot presented in Fig. 2b (Top). [file 12870_2020_2361_MOESM3_ESM.jpg]

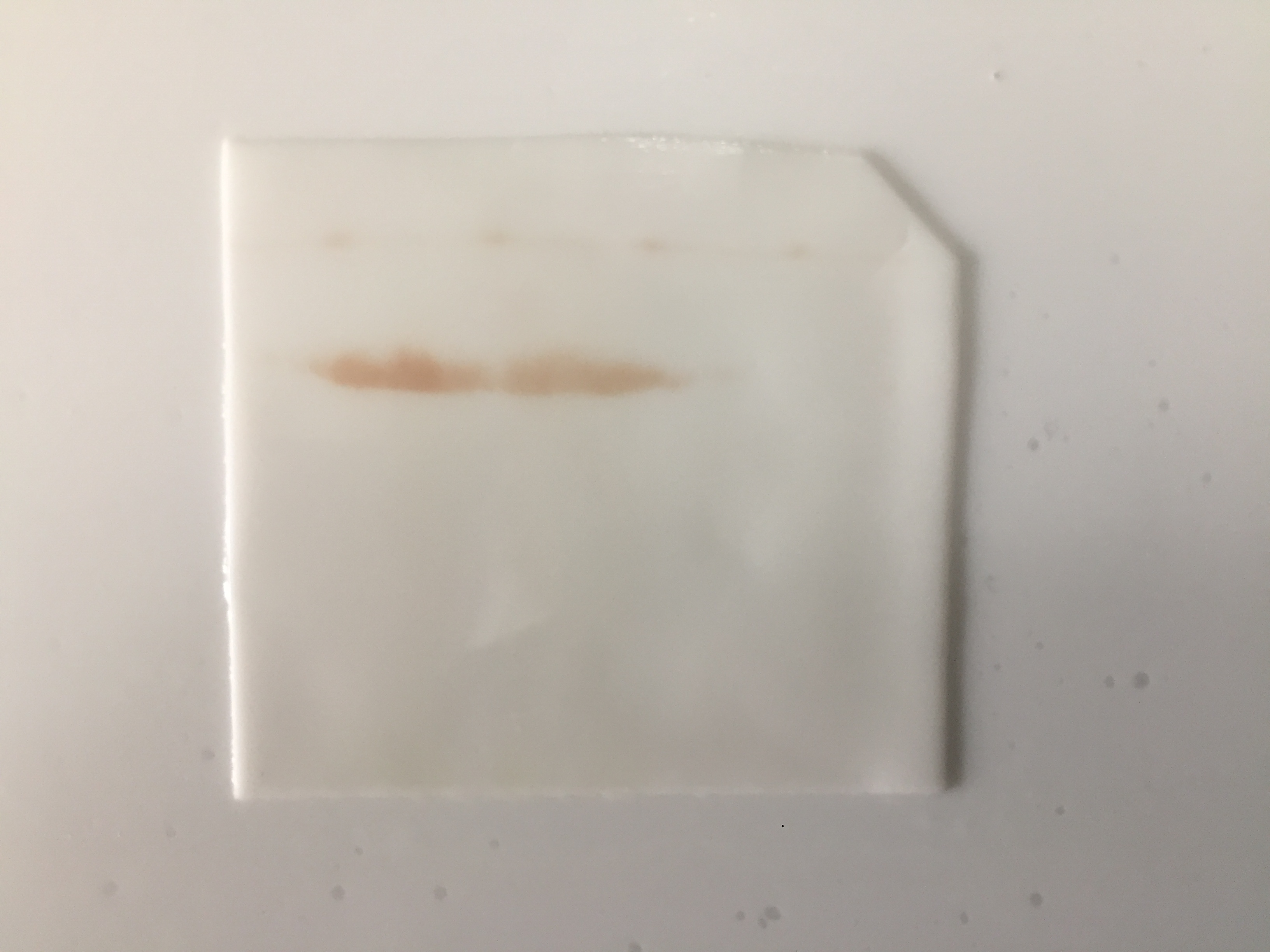

Supplement: Supplementary file 4 — Additional file 4: Figure S4. The original blot presented in Fig. 2b (Middle). [file 12870_2020_2361_MOESM4_ESM.jpg]

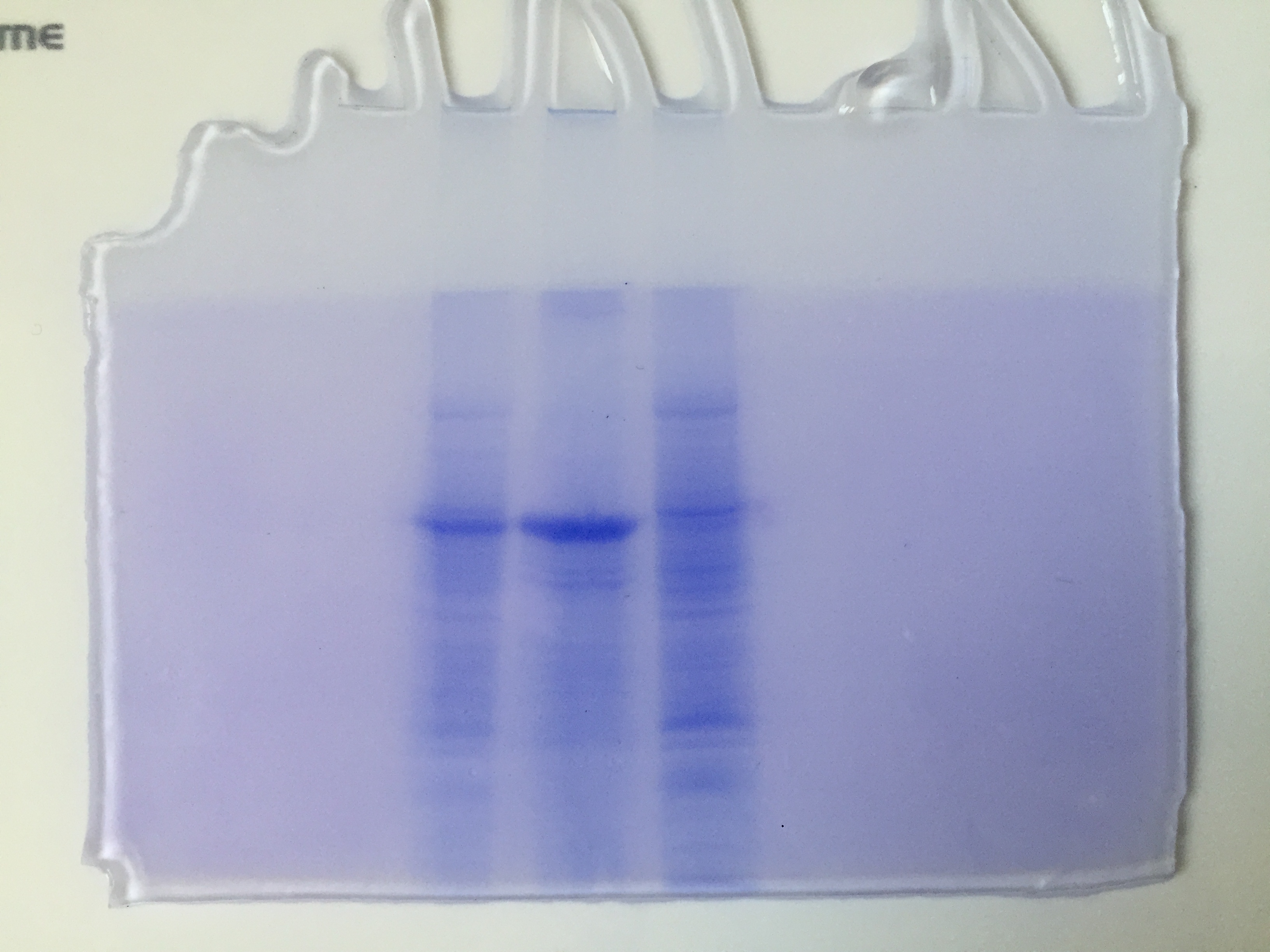

Supplement: Supplementary file 5 — Additional file 5: Figure S5. The original blot presented in Fig. 2c (Bottom). [file 12870_2020_2361_MOESM5_ESM.jpg]

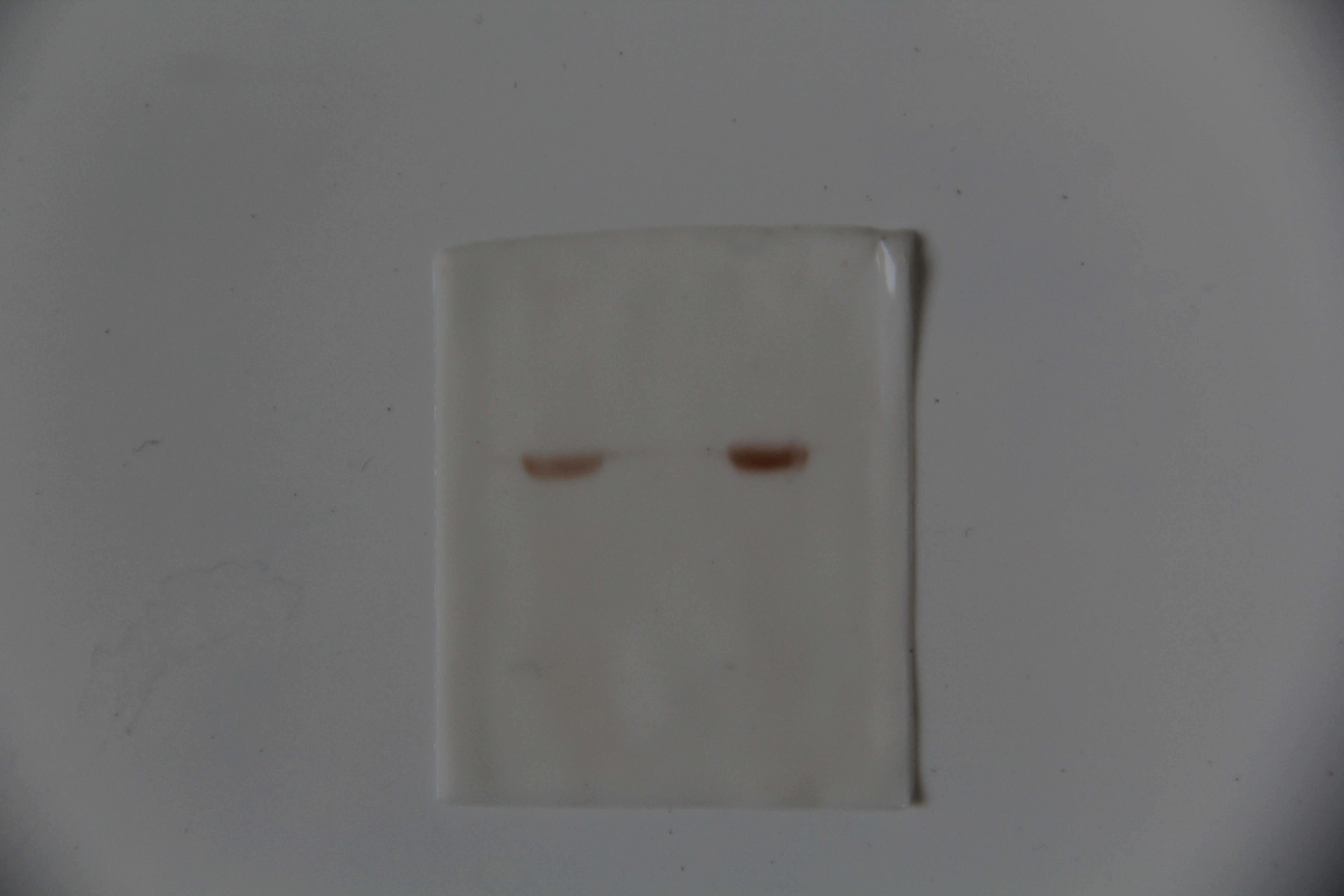

Supplement: Supplementary file 6 — Additional file 6: Figure S6. The original blot presented in Fig. 2c (Top). [file 12870_2020_2361_MOESM6_ESM.jpg]

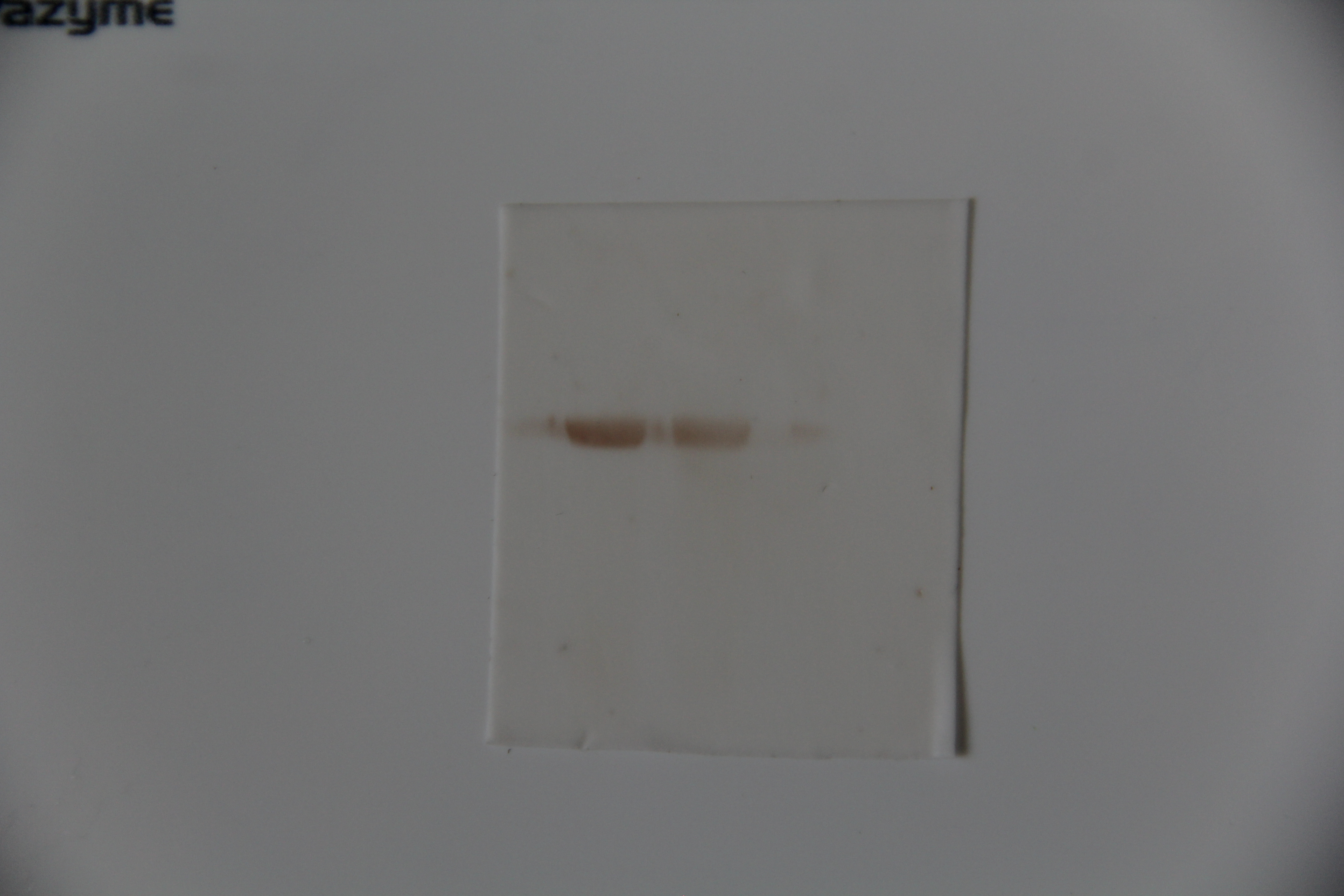

Supplement: Supplementary file 7 — Additional file 7: Figure S7. The original blot presented in Fig. 2c (Bottom). [file 12870_2020_2361_MOESM7_ESM.jpg]

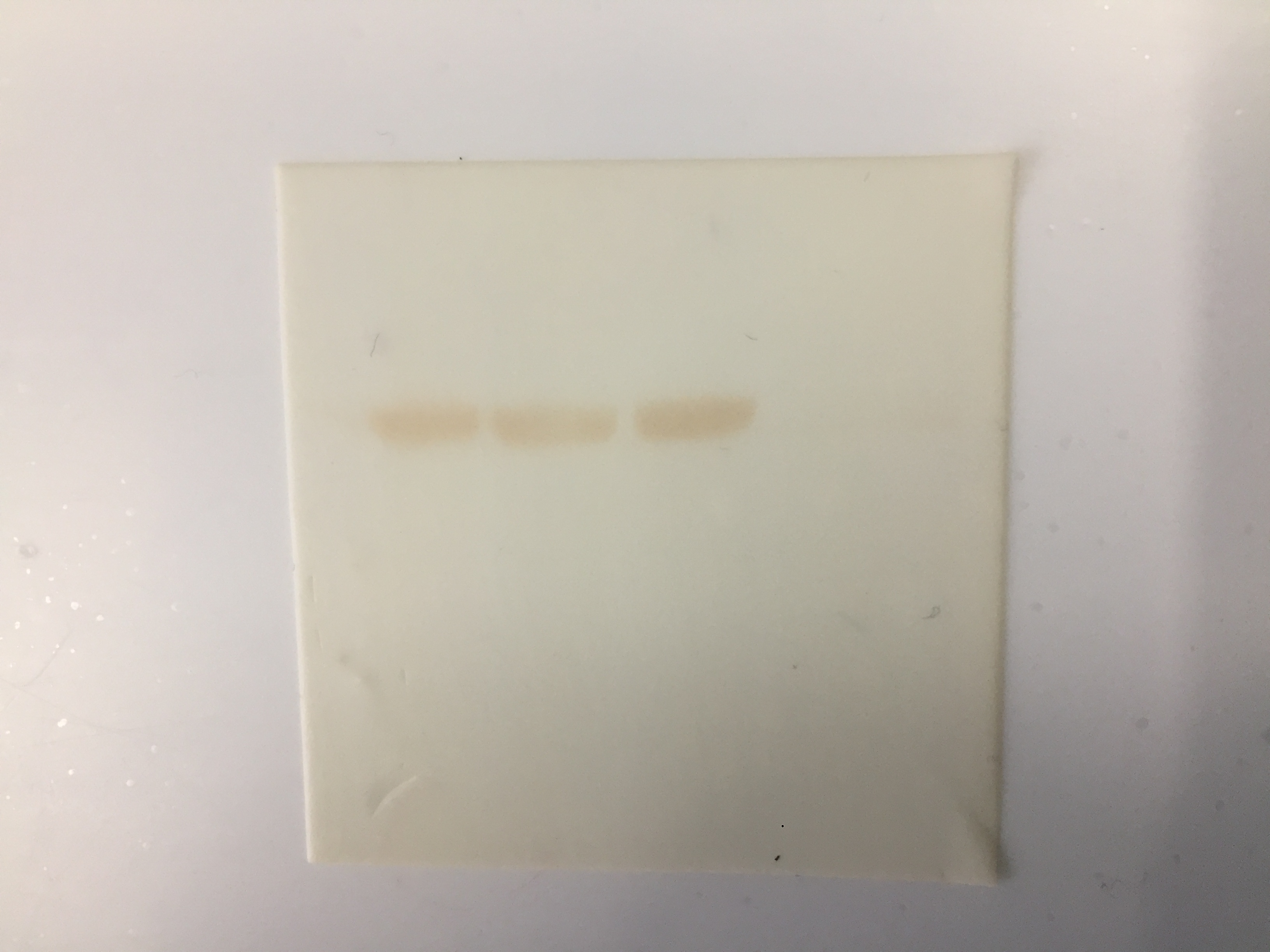

Supplement: Supplementary file 8 — Additional file 8: Figure S8. The original blot presented in Fig. 4b (Top). [file 12870_2020_2361_MOESM8_ESM.jpg]

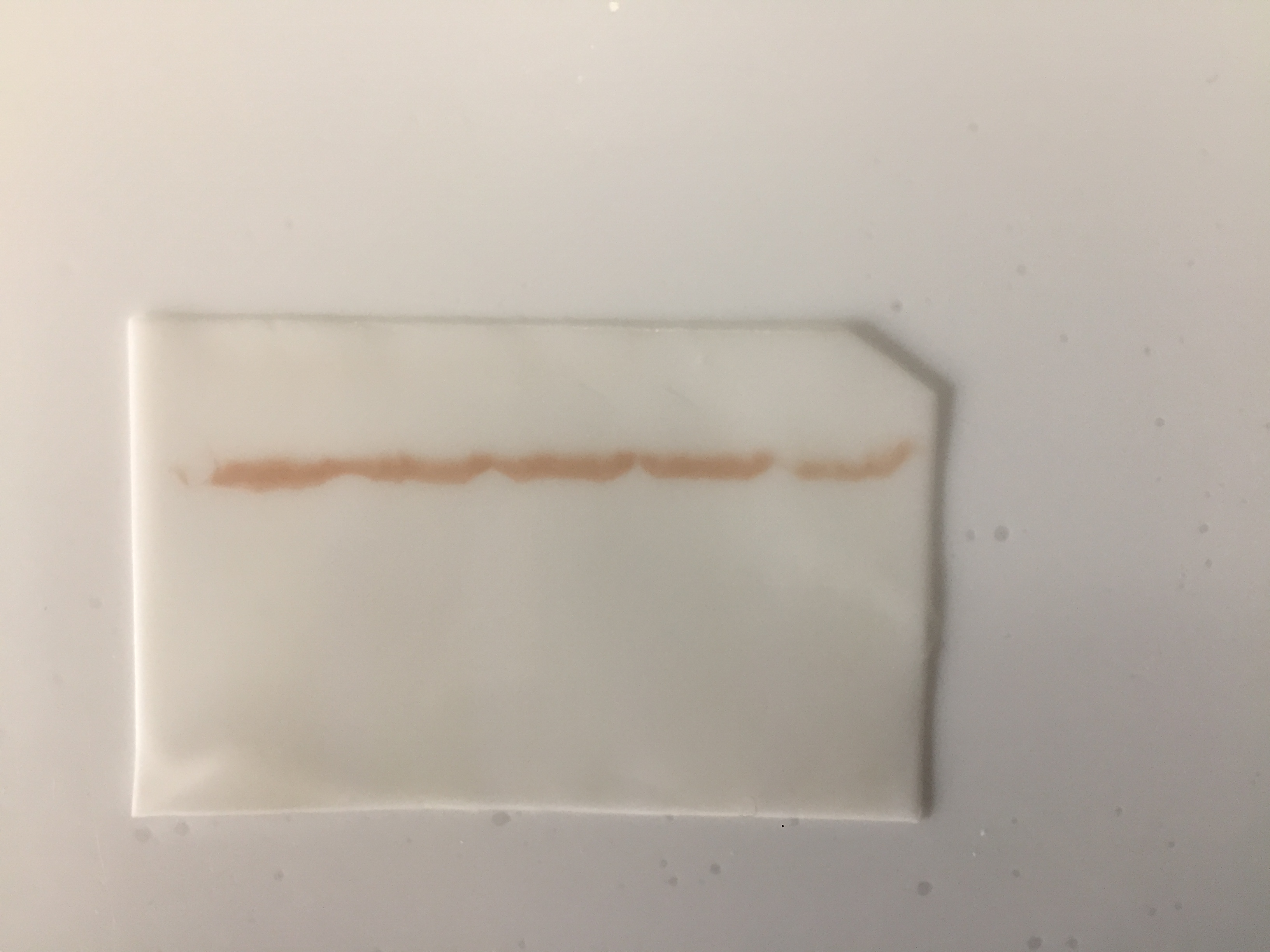

Supplement: Supplementary file 9 — Additional file 9: Figure S9. The original blot presented in Fig. 4b (Bottom). [file 12870_2020_2361_MOESM9_ESM.jpg]
