# Supplementary material for: Overexpression of an apple LysM-containing protein gene, MdCERK1–2, confers improved resistance to the pathogenic fungus, Alternaria alternata, in Nicotiana benthamiana
Source: BMC Plant Biol. 2020 Apr 8;20:146. doi: 10.1186/s12870-020-02361-z (PMC7386173; doi:10.1186/s12870-020-02361-z)
Supplement: Supplementary file 10 — Additional file 10: Table S1. The genes used in the present study. [file 12870_2020_2361_MOESM10_ESM.docx]

**Table S1*. The genes used in the present study**

| **Name** | **Species** | **Accession** |
| --- | --- | --- |
| *AtCERK1* | *A. thaliana* | At3g21630 |
| *AtLYK2* | *A. thaliana* | At3g01840 |
| *At**LYK3* | *A. thaliana* | At1g51940 |
| *AtLYK4* | *A. thaliana* | At2g23770 |
| *AtLYK5* | *A. thaliana* | At2g33580 |
| *AtLYM1* | *A. thaliana* | At1g21880 |
| *AtLYM2* | *A. thaliana* | At2g17120 |
| *AtLYM3* | *A. thaliana* | At1g77630 |
| *OsCERK1* | *O. sativa* | Os08g0538300 |
| *OsCEBiP* | *O. sativa* | Os03g0133400 |
| *OsLYP4* | *O. sativa* | Os09g27890 |
| *OsLYP6* | *O. sativa* | Os06g10660 |
| *MdCERK1-2* | *Malus domestica* | MD17G1102100 |
| *MdCERK1* | *Malus domestica* | MD09G1111800 |
| *MdLYK3* | *Malus domestica* | MD05G1351500 |
| *MdLYK4* | *Malus domestica* | MD17G1183700 |
| *MdLYK5* | *Malus domestica* | MD04G1238700 |
| *NbNPR1* | *N. benthamiana* | Niben101Scf14780g01001.1 |
| *NbPR1a* | *N. benthamiana* | Niben101Scf13926g01014.1 |
| *NbLOX1* | *N. benthamiana* | Niben101Scf01434g03006.1 |
| *NbERF1* | *N. benthamiana* | Niben101Scf00454g04003.1 |
| *NbPAL* | *N. benthamiana* | Niben101Scf02432g00011.1 |

* The gene sequences of apple, Arabidopsis, *N. benthamiana* and rice were retrieved from genomic database of apple (<https://iris.angers.inra.fr/gddh13/> ), *Arabidopsis* (<http://www.arabidopsis.org/> )*,* *N. benthamiana* (<https://solgenomics.net/tools/blast/?db_id=266>) and rice (<http://www.ricedata.cn/gene/>), respectively. The gene sequences of *GmNFR1*, *GmNFR5* and *MtLYK3* were retrieved from NCBI.
