# Supplementary material for: Overexpression of an apple LysM-containing protein gene, MdCERK1–2, confers improved resistance to the pathogenic fungus, Alternaria alternata, in Nicotiana benthamiana
Source: BMC Plant Biol. 2020 Apr 8;20:146. doi: 10.1186/s12870-020-02361-z (PMC7386173; doi:10.1186/s12870-020-02361-z)
Supplement: Supplementary file 11 — Additional file 11: Table S2. The primers used in this study. [file 12870_2020_2361_MOESM11_ESM.docx]

**Table S2. The primers used in this study**

| **Primer name** | **Primer sequence (5'→3')** |
| --- | --- |
| The full-length cDNA primers | |
| FL-*MdCERK1-2*-F | ATGGGATTTCGAATCGGGTT |
| FL-*MdCERK1-2*-R | CTACCTTCCGGACATCAGATTG |
| Primers used in constructing expressin vector | |
| *MdCERK1-2*-KpnI-F | *GGATCC*ATGGGATTTCGAATCG |
| *MdCERK1-2*-SaII-R | *GAGCTC*CTACCTTCCGGACATC |
| *MdCERK1-2-ECD*-F | *CCATGG*AGTGCAGCAAGAGCGG |
| *MdCERK1-2-ECD*-R | *CTCGAG*GGCTCCAACTTTCAGTCCTG |
| *AtCERK1-ECD*-F | *CCATGG*AGTGCAGGACTAGCTGTCC |
| *AtCERK1-ECD*-R | *CTCGAG*ACCAACACCATCTTGTTTAC |
| *AtLYM1- ECD*-F | *CCATGG*AAATCCCAGAAAAACCCA |
| *AtLYM1- ECD*-R | *CTCGAG*AGACGAAGATTAAGAAGATG |
| Quantitative real-time PCR primers | |
| *MdCERK1-2*-F | GGATGGCTCGAGTTCCGAAT |
| *MdCERK1-2*-R | TCGATCGCATACTTGGACGG |
| *MdCERK1*-F | TTTGAGGAGGCTTTATTTTC |
| *MdCERK1*-R | CCACTGGCATCTGAACATGA |
| *MdLYK3*-F | CAACGCCTTGGCCTCATACT |
| *MdLYK3*-R | TGTGTACTCGAAGGTGTGCC |
| *MdLYK4*-F | CCATTGCCGTGGTCAACAAG |
| *MdLYK4*-R | TGCCTACAAGACGAGTCTGC |
| *MdLYK5*-F | GCCACCATCGTCACCTACAA |
| *MdLYK5*-R | GTCCTCCACCGTCGTCAAAT |
| *Mdactin*-F | TGCTGTAAATATGTGGGTTAATGAG |
| *Mdactin*-R | GAAATTGCCTGGAGGAGAATAG |
| *NbNPR1*-F | GGCGAGGAGTCCGTTCTTTAA |
| *NbNPR1*-R | TCAACCAGGAATGCCACAGC |
| *NbPR1a*-F | AACCTTTGACCTGGGACGAC |
| *NbPR1a*-R | AAGTGTTTGAGTCGTGGGCA |
| *NbLOX1*-F | GTTGAAGGTTCTATCTGGCAGTTGG |
| *NbLOX1*-R | TGTTGCGATCACGAATGGCTCTA |
| *NbERF1*-F | GGCGAATTTTCCGGGAGACT |
| *NbERF1*-R | GGCTCCGATTTTACTTCGCC |
| *NbPAL4*-F | GGGCAGCTATGTTAGTTAGGATCAACGG |
| *NbPAL4*-R | GGCAAACATGGAGTAACATTGTGGT |
| *Nbactin*-F | AGGAGAAGTTGGCTTACATTGC |
| *Nbactin*-R | CCGATCATTGATGGTTGGAACA |
